# Full function of exon junction complex factor, *Rbm8a*, is critical for interneuron development

**DOI:** 10.1038/s41398-020-01065-0

**Published:** 2020-11-05

**Authors:** Colleen McSweeney, Fengping Dong, Miranda Chen, Jessica Vitale, Li Xu, Nicole Crowley, Bernhard Luscher, Donghua Zou, Yingwei Mao

**Affiliations:** 1 Department of Biology, Pennsylvania State University, University Park, PA 16802 USA; 2 Zhejiang Chinese Medical University, 310053 Hangzhou, Zhejiang China; 3 Department of Biochemistry and Molecular Biology, Pennsylvania State University, University Park, PA 16802 USA; 4 Department of Neurology, The Fifth Affiliated Hospital of Guangxi Medical University, 530021 Nanning, Guangxi China

**Keywords:** Stem cells, Molecular neuroscience

## Abstract

The formation of the nervous system requires a balance between proliferation, differentiation, and migration of neural progenitors (NPs). Mutations in genes regulating development impede neurogenesis and lead to neuropsychiatric diseases, including autism spectrum disorders (ASDs) and schizophrenia (SZ). Recently, mutations in nonsense-mediated mRNA decay genes have been associated with ASDs, intellectual disability (ID), and SZ. Here, we examine the function of a gene in the exon junction complex, *Rbm8a*, in the cortical development. When *Rbm8a* is selectively knocked out in neural stem cells, the resulting mice exhibit microcephaly, early postnatal lethality, and altered distribution of excitatory neurons in the neocortex. Moreover, *Rbm8a* haploinsufficiency in the central nervous system decreases cell proliferation in the ganglionic eminences. Parvalbumin+ and neuropeptide Y+ interneurons in the cortex are significantly reduced, and distribution of interneurons are altered. Consistently, neurons in the cortex of conditional knockout (cKO) mice show a significant decrease in GABA frequency. Transcriptomic analysis revealed differentially expressed genes enriched in telencephalon development and mitosis. To further investigate the role of *Rbm8a* in interneuron differentiation, conditional KO of *Rbm8a* in NKX2.1 interneuron progenitor cells reduces progenitor proliferation and alters interneuron distributions. Taken together, these data reveal a critical role of *Rbm8a* in interneuron development, and establish that perturbation of this gene leads to profound cortical deficits.

## Introduction

The development of the cortex is a delicate balance between proliferation, differentiation, and migration of neural progenitors (NPs). Throughout developmental process, various cellular mechanisms ensure that NPs differentiate into the correct cell subtypes, migrate to their correct regions, and form the correct cortical layers. The cortex is comprised of both excitatory and inhibitory neurons, which interact within neuronal circuits to mediate cortical functions. Though both types of neurons reside in the cortex, they arise from different embryonic brain regions, and from different NPs. Excitatory neurons are generated from NPs residing in the ventricular zone (VZ)/subventricular zone (SVZ) of the lateral ventricle. These progenitors undergo asymmetric division to concurrently renew themselves, and to generate the immature neurons that will migrate up the radial glia and differentiate into excitatory neurons in the cortex. Conversely, inhibitory interneurons arise from the ganglionic eminence (GE), comprised of the lateral, caudal, and medial ganglionic eminence (LGE, CGE, and MGE respectively), and undergo tangential migration to reach the cortex. The MGE specifically is where most interneuron progenitors originate, namely the most populous cortical interneuron, parvalbumin (PV), and somatostatin (SST)-positive interneurons. Neuropeptide Y (NPY) and calretinin (CR) interneurons all originate from a combination of the MGE and CGE^1^.

Interneuron progenitors are DLX1/2 positive and interneuron progenitors arising from the MGE are positive for NKX2.1^1^. Interneurons can be classified according to a variety of properties, such as morphology, molecular markers, and physiological capabilities. NKX2.1 progenitors produce several interneuron subtypes, PV, SST, and NPY^2^. PV interneurons can be subdivided into basket and chandelier cells, both of which are fast spiking. SST cells can be divided into martinotti cells and X94 cells. X94 cells are located exclusively in layers 4 and 5, and martinotti cells can be either CR positive or negative, and are bursting/non-bursting, respectively. NPY neurons originate from both the MGE and CGE, and can also be SST positive. In the adult mouse brain, they are found mostly in layers 2/3/6, but can exist in all layers^3^. In addition, differentiation of interneuron subtype is often activity dependent^4^.

Cortical developmental deficits have been observed in patients with developmental disorders, such as autism spectrum disorders (ASDs) and schizophrenia (SZ)^5^. Though less populous than excitatory neurons in the neocortex, GABAergic interneurons are essential to the functionality of the nervous system. Increasing evidence show that deficits in interneurons are related to psychiatric disease. Postmortem studies of SZ patients revealed reduced GAD67 expression, and decreased *PV* and *GAD67* mRNA^6,7^. In addition, 30% of patients with ASD have epilepsy, and 30% of patients with epilepsy have ASD^8^. This comorbidity suggests a potential disruption in the excitatory/inhibitory (E/I) balance of the brain, which often results in epilepsy. In addition, many ASD risk genes have been associated with GABAergic signaling. For example, deficits in CNTNAP2 also lead to decreased number of PV+ interneurons^9^. *DISC1* gene has been associated with SZ, depression, and bipolar disorder. *DISC1* mouse models showed various defects in interneurons^10,11^. Taken together, these results provide some evidence for the GABAergic hypothesis of ASD.

Recently, rare variants of genes in nonsense-mediated mRNA decay (NMD) have been associated with mental diseases. NMD identifies and tags mRNAs with pretermination stop codons (PTCs) for degradation. A NMD factor, *UPF3B* has linked to ASD, early onset SZ, and intellectual disability (ID)^12–14^. In addition, NMD relies on the exon junction complex (EJC) to define the RNA substrates with PTCs for decay. One prominent copy number variation associated with neurodevelopmental disorders is 1q21^15^. *RBM8A*, a core member of the EJC, is localized in the distal region of 1q21. Compound mutations of *RBM8A* cause thrombocytopenia with absent radius (TAR) syndrome^16^, a disorder characterized by missing radius bones, low blood platelet counts, and ID (in 7% of cases). Previous studies showed that *Rbm8a* modulates the balance between the proliferation and differentiation of NPs^17,18^. Increase of RBM8A expression leads to more cells proliferating in the VZ/SVZ and fewer cells migrating to the cortical plate (CP). Conversely, knockdown (KD) or knockout (KO) of *Rbm8a* leads to less cells remaining in the VZ/SVZ and more cells migrating to the CP^17,18^.

Our previous studies demonstrated that RBM8A modulates animal behaviors^19^, and overexpression of RBM8A increases expression of GABA receptors GABRG3, GABRB3, and GABRP^17^. However, very little is known about how *Rbm8a* affects GABAergic neuron development in animals. Here, we established a conditional KO (cKO) mouse line in *Rbm8a* locus. By crossing with Nestin-cre mouse line, *Rbm8a* haploinsufficiency in the nerve system leads to significantly smaller brains than littermate controls. Migration and cell size of cortical interneurons are abnormal. Consistently, *Rbm8a* haploinsufficiency in NKX2.1-positive interneuron progenitor decreases cell proliferation and affects interneuron differentiation. Thus, our observations reveal an important role of *Rbm8a* in interneuron development.

## Results

### KO of *Rbm8a* in the mouse brain

Our previous study has pointed out a vital role of *Rbm8a* in brain development. To further study how *Rbm8a* regulates brain development, we developed a cKO mouse. A targeting vector was designed with loxp sites flanking exons 2–4, and a neomycin-resistance cassette (PGK-neo) inserted downstream of exon 4 inside the loxP sites (Supplemental Fig. S1A). To avoid the potential unexpected effects of PGK-neo insertion, the neo cassette was removed by crossing with Actin-Flp transgenic mice. When exposed to cre DNA recombinase, exons 2–4 will be deleted resulting in a frame shift that causes a premature nonsense mutation. The truncated mRNA is prone to degradation via NMD or produces a nonfunctional protein. The presence of the loxp/frt sites was confirmed via PCR (Supplemental Fig. S1B). Long-range PCR confirmed the correct targeting of 5′ and 3′ homolog arms (Supplemental Fig. S1C). To test the effectiveness of our cKO strategy, fibroblast cells from homozygous *Rbm8a*^*fl/fl*^ mice were infected with an RFP-cre virus in vitro. RBM8A protein was absent in RFP-positive cells (cre+, white arrowheads), but still expressed in RFP-negative cells (blue arrowheads; Supplemental Fig. S1D). In addition, when using saturated cre virus, the lysate from infected fibroblast cells was collected, and Western blot confirmed the absence of RBM8A protein (Supplemental Fig. S1E). These data confirmed that we successfully knocked out *Rbm8a* in mice.

### *Nes-cre; Rbm8a*^*fl/+*^ mice show microcephaly and abnormal development

**Fig. 1:**
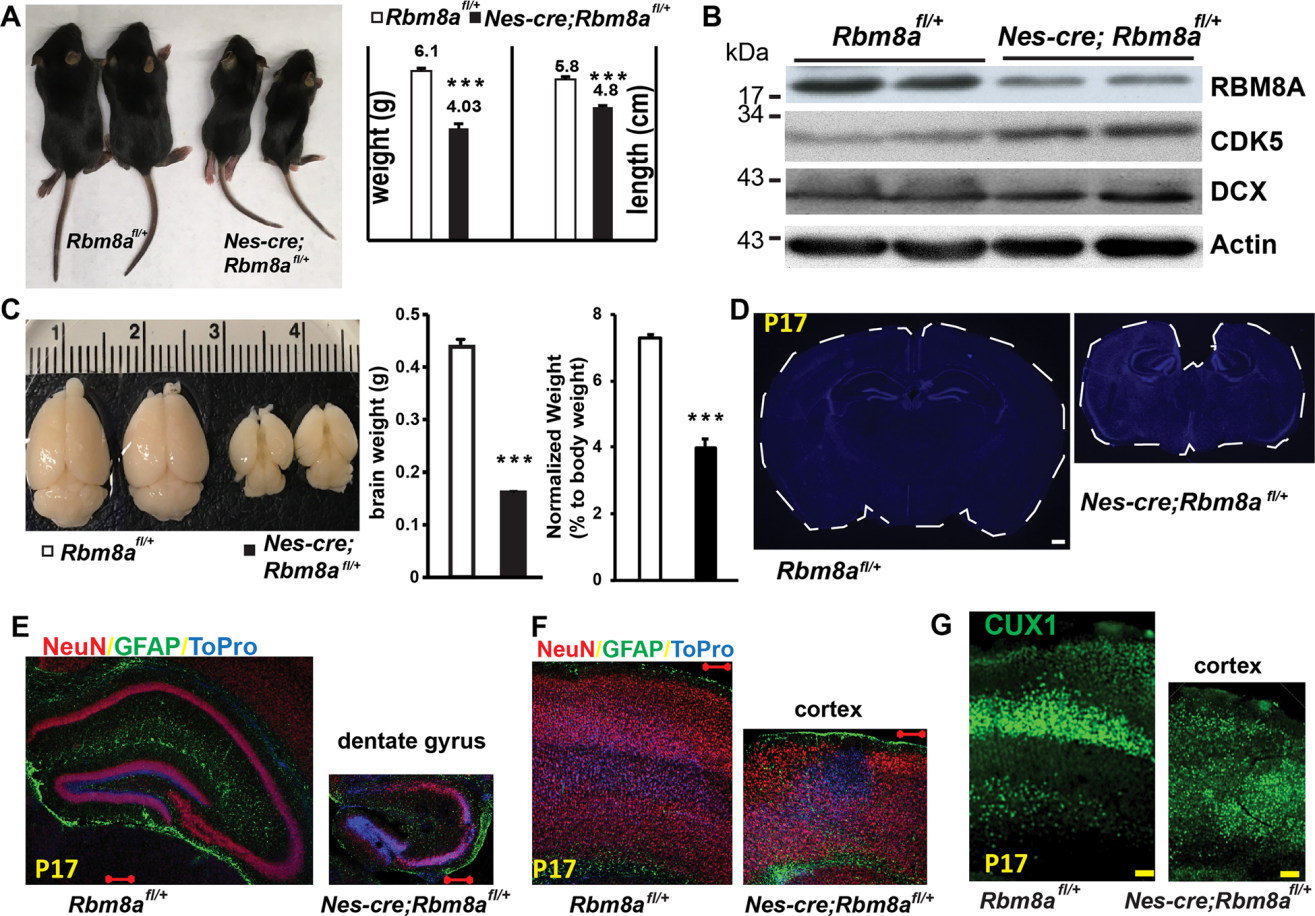
*Rbm8a* haploinsufficiency modulates brain size. **A** *Nes-cre; Rbm8a*^*fl/+*^ mice are smaller than littermate controls at P17 in both body weight and length. ***p = 5.4 × 10^−5^ (weight), *p* = 1.4 × 10^−4^ (length), *n* = 3, Student’s *t* test. **B** *Nes-cre; Rbm8a*^*fl/+*^ mice show an ~50% reduction in RBM8A protein expression in the brain, as determined by Western blot. They have ~50% more CDK5 protein expression. **C** *Nes-cre; Rbm8a*^*fl/+*^ mice at P17 suffer from microcephaly (small brain), even when normalizing for their smaller body size. A ruler is included to show the actual brain size. ***p = 1.9 × 10^−5^ for brain weight and *p* = 2.6 × 10^−4^ for normalized brain weight, *n* = 3, Student’s *t* test. **D** *Nes-cre; Rbm8a*^*fl/+*^ mice have a smaller brain than *Rbm8a*^*fl/+*^ controls, and have cortical hemispheres that fail to meet at the midline, causing a large gap between the two cortical hemispheres. DAPI staining of coronal brain slices at P17. Scale bar = 50 µm. **E** P17 hippocampus of *Nes-cre; Rbm8a*^*fl/+*^ mice and littermate controls stained for mature neuronal neuronal marker NeuN (red), reactive astrocyte marker GFAP (green), and DNA marker DAPI (blue). **F** P17 cortex stained for NeuN (red), GFAP (green), and DAPI. *Nes-cre;* *Rbm8a*^*fl/+*^ mice have a thinner cortex and less cell dense cortex. **G** Coronal sections of P17 *Nes-cre; Rbm8a*^*fl/+*^ and *Rbm8a*^*fl/+*^ mice brains were immunostained for the superficial layer marker CUX1. Scale bar, 100 µm. *Nes-cre; Rbm8a*^*fl/+*^ mice had an abnormal distribution CUX1 (stains deep layers in the medial cortex).

Next, we crossed our homozygous floxed mice with nestin-cre (*Nes-cre*) mice to generate heterozygous *Rbm8a* cKO selectively in NPs. Intriguingly, *Nes-cre*; *Rbm8a*^*fl/+*^ mice were significantly smaller and weighed significantly less than *Rbm8a*^*fl*/+^ littermate controls (Fig. 1A). Western blot analysis using brain lysate from *Nes-cre*; *Rbm8a*^*fl/+*^ and *Rbm8a*^*fl/+*^ at postnatal day 14 (P14) showed a twofold decrease in RBM8A expression, as the mice are heterozygous (Fig. 1B). An increase in CDK5 and DCX expression indicates the elevated premature neuronal differentiation reported previously^17^. *Nes-cre*; *Rbm8a*^*fl/+*^ mice suffered from microcephaly even when normalizing for small body size (Fig. 1C, D). NeuN staining revealed the abnormal morphology of the hippocampus (HP), characterized by a dentate gyrus (DG) that points dorsally, a rounded CA3 region, and less cell density in the CA1 (Fig. 1E). In addition, the cortex was much thinner (Fig. 1F) and, less cell dense (Supplemental Fig. S2). Consistently, we found that cortical neuron marker, CUX1, is clearly enriched in cortical layers 2–3 of *Rbm8a*^*fl/+*^ mice, but distributed throughout all different layers in *Nes-cre*; *Rbm8a*^*fl/+*^ mice (Fig. 1G), suggesting the loss of the cortical lamination.

The other organs in the *Nes-cre; Rbm8a*^*fl/+*^ mice were weighed and compared to the littermate controls. Besides the brain, the heart, kidneys, and spleen were significantly smaller in the *Nes-cre; Rbm8a*^*fl/+*^ mice (Supplemental Fig. S3A). One explanation of the smaller brain could be a proportional reduction due to a smaller whole body size in the cKO mouse. However, when the organ weights were normalized to the body weight of the mouse, there was no significant difference in the weight of the other organs of the *Nes-cre; Rbm8a*^*fl/+*^ mice (Supplemental Fig. S3B). These data support that the smaller brain in cKO mouse is not just a proportional reduction of the body size. Thus, *Rbm8a* cKO mice show specific deficits in the nervous system.

### *Rbm8a* haploinsufficiency changes the proliferation of neural progenitors in the GE

**Fig. 2:**
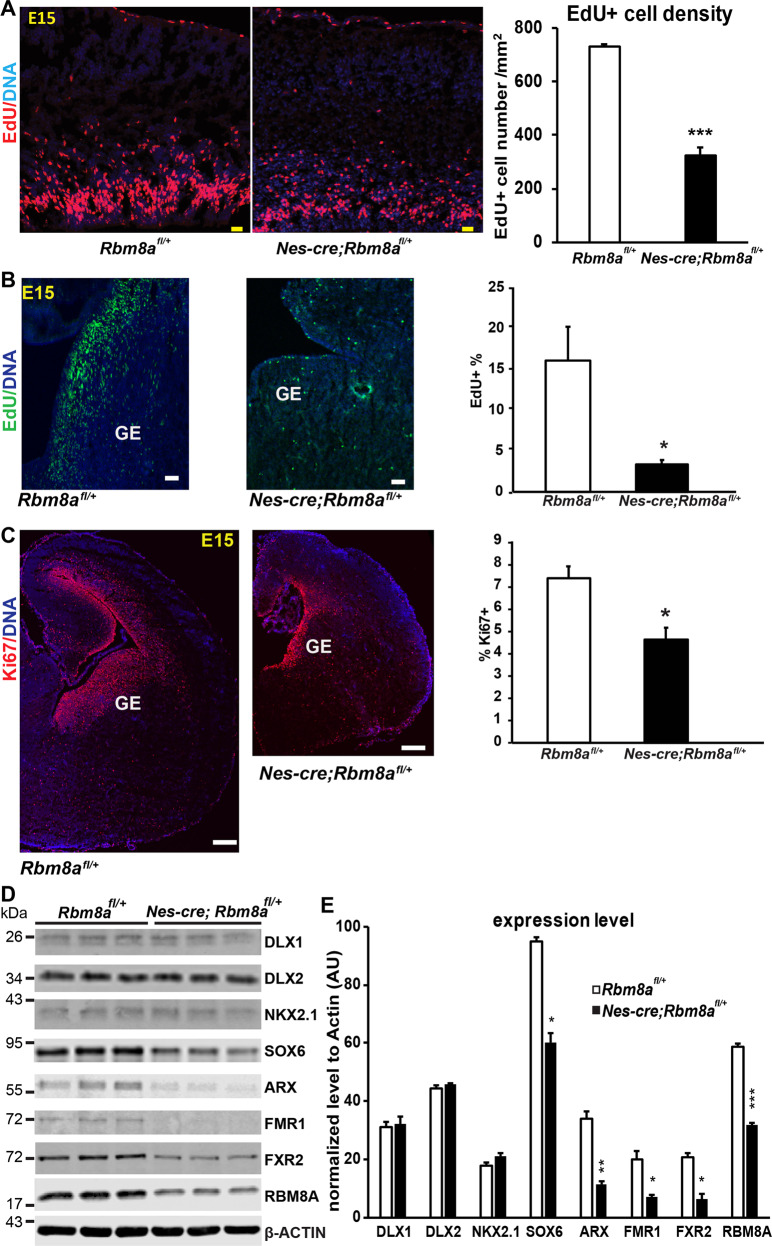
*Rbm8a* haploinsufficiency decreases the proliferation of cells in the neocortex and GE. **A** Pregnant dams were injected with EdU 1 h prior to dissection of E15 embryos. Embryos were then stained for EdU using click chemistry. *Nes-cre; Rbm8a*^*fl/+*^ mice, the cortical NPs were significantly decreased in E15 *Nes-cre; Rbm8a*^*fl/+*^ mice. The number of EdU+ cells were quantified in the neocortex and divided by area (mm^2^). Scale = 30 µm. *p < 0.05; **p < 0.01; *n* = 3, Student’s *t* test. **B** *Nes-cre; Rbm8a*^*fl/+*^ mice have significantly fewer cells in the DNA synthesis phase of the cell cycle in the GE. The number of EdU+ cells were quantified in the GE, and divided by the total number of DAPI+ cells and multiplying by 100 to get the percentage of EdU+ cells, *p = 0.01, *n* = 3, Student’s *t* test. Scale = 50 µm. **C** *Nes-cre; Rbm8a*^*fl/+*^ mice have significantly fewer cells active in the cell cycle (non-quiescent, undifferentiated). E15 brains were stained with cell cycle marker anti-Ki67 antibody. The number of Ki67+ cells were quantified in the GE then divided by the total number of DAPI+ cells and multiplying by 100 to determine the percentage of Ki67+ cells, *p = 0.02, *n* = 3, Student’s *t* test. **D** Western blots showed significant reduction in ARX, FMR1, and FXR2 protein expression in *Nes-cre; Rbm8a*^*fl/+*^ brain. E. Quantification of protein levels in Western blots of 2D, normalized with ACTIN. *p < 0.05; **p < 0.01; ***p < 0.001; *n* = 3, Student’s *t* test.

To further test if microcephaly and laminar disorganization are caused by NP defects, we injected 10 mg/kg EdU to label proliferating NPs during S phase. Consistent with our previous study^17^, cortical NP proliferation was decreased over 50% in *Nes-cre*; *Rbm8a*^*fl/+*^ mice (Fig. 2A). In addition to reduced cell proliferation, apoptosis was increased in the embryonic cortex at E16 (Supplemental Fig. S4A). Interestingly, most apoptotic cells are localized in the intermediate zone and CP, suggesting that dying cells are likely to be immature neurons. These data supported that *Rbm8a* cKO lead to defects in cell proliferation and death. As nestin expression can be detected in the GE region^11^, we further tested if the progenitors in the GE are affected by *Rbm8a* deficiency. We detected over threefold decrease of cells in the S phase of the cell cycle in the GE of *Nes-cre*; *Rbm8a*^*fl/+*^ mice (Fig. 2B). The cells at S phase of the cell cycle in the *Nes-cre*; *Rbm8a*^*fl/+*^ mice were less dense than control (Fig. 2B). Consistently, Ki67-positive cells were reduced ~30% to the control litter mates (Fig. 2C).

Multiple transcriptional factors, such as DLX1/2, NKX2.1, SOX6, and ASD risk genes, including ARX and FMR1, are involved in interneuron differentiation^20^. We further tested the protein expression of DLX1, DLX2, NKX2.1, SOX6, and ARX, FMR1, FXR2 in our mouse model (Fig. 2D, E). Interestingly, SOX6 showed over 30% reduction and ARX, FMR1, and FXR2 showed over 60% decrease of protein levels in cKO mice (Fig. 2D, E). Although we detected significantly change of progenitor proliferation in the GE area, the expression levels of some key transcriptional factors controlling interneuron progenitors, such as DLX1/2 and NKX2.1, remained unchanged (Fig. 2D, E). In order to assess the number and distribution of these progenitors, we further stained the embryonic brains for interneuron progenitor marker NKX2.1. Consistent with the western blot result, cell density of NKX2.1+ cells in the GE at E15 were slightly decreased, but the distribution of NKX2.1+ cells were affected (Supplemental Fig. S4B). These results support that *Rbm8a* is required for proper interneuron progenitor proliferation and differentiation.

### *Rbm8a* cKO altered number and distribution of interneurons in the cortex

**Fig. 3:**
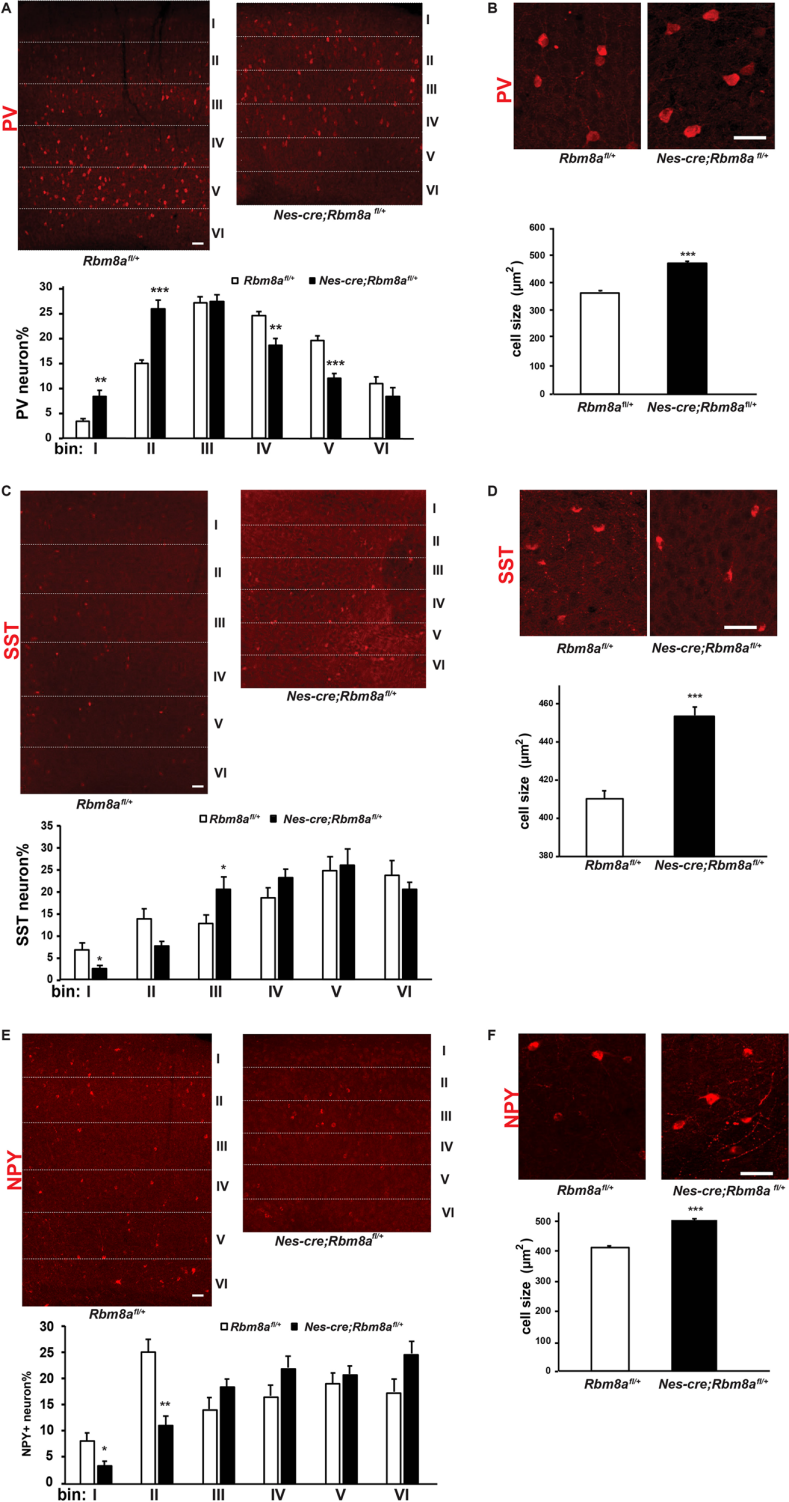
*Rbm8a* regulates the generation and migration of PV+, SST+, and NPY+ interneurons. **A** Cell number of PV+ interneurons was decreased, and the distribution of PV+ interneurons was altered in the cortex of *Nes-cre; Rbm8a*^*fl/+*^ mice. Scale bar = 50 µm. Quantification of the distribution of PV+ interneurons in the cortex. There are significantly altered PV+ interneurons in bins I, II, IV, and V compared to *Rbm8a*^*fl/+*^ mice (**p < 0.01, ***p < 0.0001; *t* test, *n* = 3). **B** PV+ cells in *Nes-cre; Rbm8a*^*fl/+*^ mice are significantly larger than in *Rbm8a*^*fl/+*^ mice. Scale bar = 50 µm. Quantification of the size of PV+ interneurons (***p < 0.001; *t* test; *δ* = 0.562; Cohens *δ*). **C** *Nes-cre; Rbm8a*^*fl/+*^ mice have altered distribution of SST+ interneurons in the cortex. Scale bar = 50 µm. Quantification of the distribution of SST+ interneurons in the cortex. There are significant changes of SST+ interneurons in bins I and III, compared to *Rbm8a*^*fl/+*^ mice (*p < 0.05; *t* test, *n* = 3). **D** SST+ cells in *Nes-cre; Rbm8a*^*fl/+*^ mice are significantly larger than in *Rbm8a*^*fl/+*^ mice. Scale bar = 50 µm. Quantification of the size of SST+ interneurons in the cortex (***p < 0.001; *t* test; *δ* = 0.359; Cohens *δ*). **E** *Nes-cre; Rbm8a*^*fl/+*^ mice showed altered distribution of NPY+ interneurons in the cortex. Scale bar = 50 µm. There are significantly fewer NPY+ interneurons in bins I and II, compared to *Rbm8a*^*fl/+*^ mice (*p < 0.05, **p < 0.01; *t* test, *n* = 3). **F** NPY+ cells in *Nes-cre; Rbm8a*^*fl/+*^ mice are significantly larger than in *Rbm8a*^*fl/+*^ mice. Scale bar, 50 µm. Quantification of the size of NPY+ interneurons in the cortex (***p < 0.001; *t* test; *δ* = 0.793; Cohens *δ*).

Since the proliferation of progenitors in the GE was significantly decreased in *Nes-cre; Rbm8a*^*fl/+*^ mice, we further hypothesized that interneuron differentiation was altered by *Rbm8a* haploinsufficiency. We assessed the number and distribution of PV+ interneurons in our *Nes-cre; Rbm8a*^*fl/+*^ mice. P17 brain slices were stained with a PV antibody, and the cell number and their distribution in the dorsal lateral prefrontal cortex (DLPFC) were quantified. *Nes-cre*; *Rbm8a*^*fl/+*^ had significantly fewer PV+ cells in the DLPFC (Supplemental Fig. S5A). To determine distribution of PV+ cells, the cortical slices were divided into six bins, each of equal size, and the number of PV+ cells was counted in each bin (Fig. 3A). Significantly, more PV+ cells were located in bins I and II of the cortex of cKO mice, but significantly fewer PV+ cells were in bins IV and V. Bins III and VI did not have any observable difference between *Nes-cre*; *Rbm8a*^*fl/+*^ and *Rbm8a*^*fl/+*^mice (Fig. 3A). The abnormalities in PV+ interneurons and the small size of the cerebral cortex led us to further investigate whether the size of PV+ interneurons was changed in the *Nes-cre*; *Rbm8a*^*fl/+*^ mice. We calculated the soma size of cells in *Nes-cre*; *Rbm8a*^*fl/+*^ mice and *Rbm8a*^*fl/+*^, and found that PV+ interneurons in *Nes-cre*; *Rbm8a*^*fl/+*^ mice were significantly larger than the control mice (Fig. 3B).

As SST+ interneurons also play important roles in the cortex, we further examined whether SST+ interneurons had an abnormal cell number and distribution. The percentage of SST+ interneurons in the cortex was unchanged, but the distribution of SST+ cells in the cortex was abnormal compared to *Rbm8a*^*fl/+*^ mice (Supplemental Fig. S5B and Fig. 3C). Significant fewer SST+ cells were in bins I and II in *Nes-cre*; *Rbm8a*^*fl/+*^, and significantly more SST+ cells were in bin III. Bins IV–VI remained unchanged (Fig. 3C). Similarly, cell size of SST+ interneurons was significantly larger in *Nes-cre*; *Rbm8a*^*fl/+*^ mice (Fig. 3D). We often detected few punctate SST stainings that are derived from dendrites in SST neurons in KO group, suggesting the dendritic development of SST neurons may be impaired (Fig. 3D).

Moreover, we examined the number and distribution of NPY+ interneurons in the cortex. Similar to PV+ interneurons, significantly fewer NPY+ interneurons were detected in the cortex of *Nes-cre*; *Rbm8a*^*fl/+*^ mice (Supplemental Fig. S5C and Fig. 3E). In addition, these interneurons were significantly less abundant in bins I and II, and unchanged in the other bins (Fig. 3E). Interestingly, the size of NPY+ interneurons was significantly larger in *Nes-cre*; *Rbm8a*^*fl/+*^ mice (Fig. 3F). Collectively, these results supported a critical role of *Rbm8a* in interneuron development.

### *Rbm8a* haploinsufficiency modulates GABA but not glutamate transmission

**Fig. 4:**
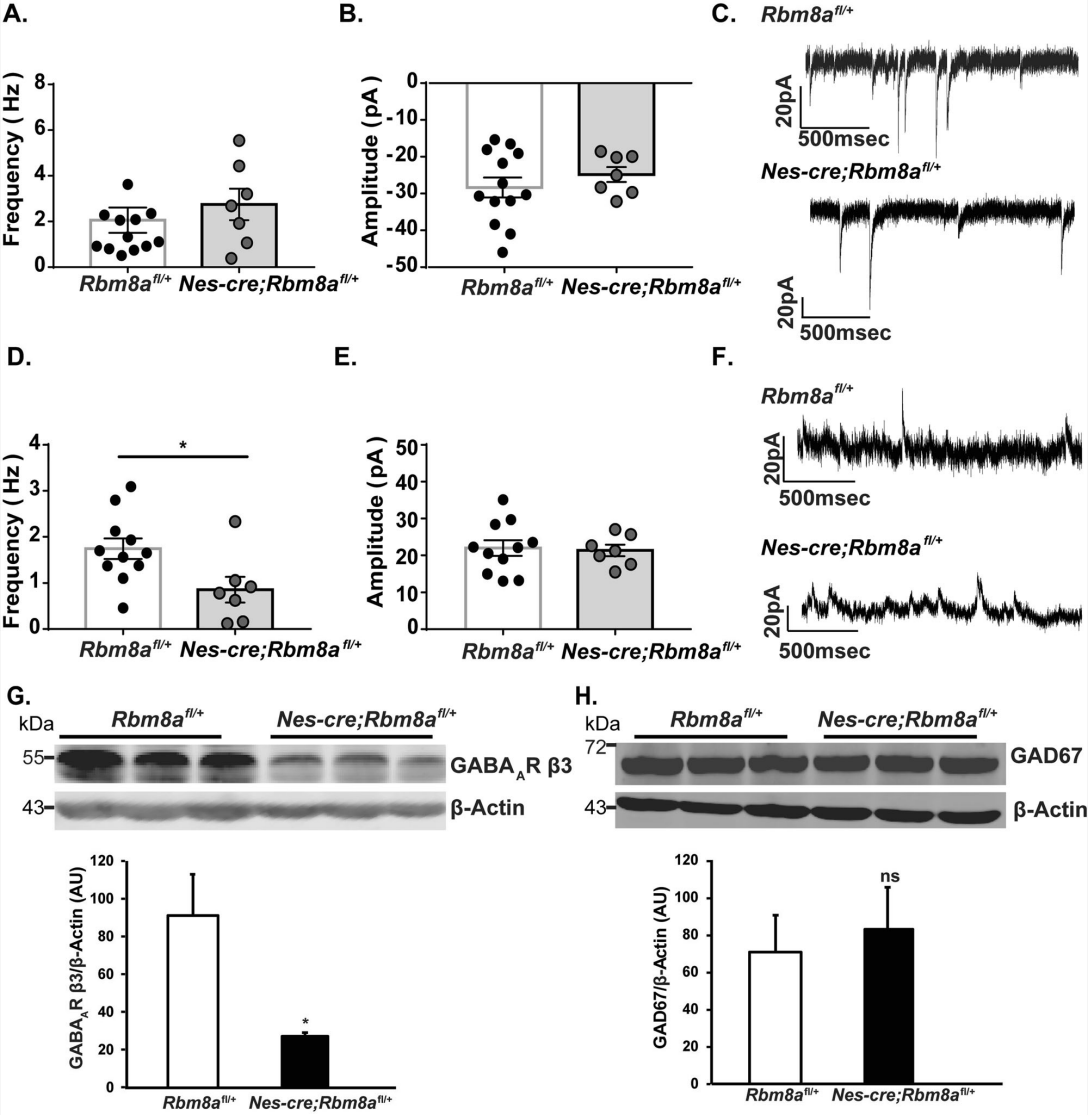
Putative pyramidal cells in the somatosensory cortex have decreased GABA amplitude. **A**, **B** There is no change in the frequency (**A**) or amplitude (**B**) of the excitatory spontaneous postsynaptic current of *Nes-cre; Rbm8a*^*fl/+*^ mice. Spontaneous excitatory postsynaptic current recordings from putative pyramidal cells in the somatosensory cortex, Student’s *t* test, *n* = 7–12. **C** Representative glutamate traces from control and cKO mice. **D**, **E** There is a significant decrease in the frequency (**D**), but not the amplitude (**E**) of the spontaneous inhibitory postsynaptic current in *Nes-cre; Rbm8a*^*fl/+*^ mice. Spontaneous inhibitory postsynaptic current recordings from putative pyramidal cells in the somatosensory cortex, *p < 0.05, *n* > 7, Student’s *t* test. **F** Representative GABA trace from control and cKO mice. Bar graphs represent means ± SEMs. **G** Western blot showed a significant reduction of GABA-A receptor β3 subunit. *p < 0.05; *n* = 3, Student’s *t* test. Bar graphs represent means ± SEMs. **H** Western blot showed a no significant reduction of GAD67. *n* = 3, Student’s *t* test. Bar graphs represent means ± SEMs.

Due to the significant perturbations in the number and distribution of interneurons in the cortex, we hypothesized that this would in turn affect the electrophysiological properties of pyramidal cells in the cortex (which form circuits with interneurons). To examine the excitatory neurotransmission, control and cKO mice were sacrificed at P14, and spontaneous transmitter recordings were taken from putative pyramidal cells in the somatosensory cortex (Fig. 4A–C). We did not observe significant changes the frequency (Fig. 4A, C) or amplitude (Fig. 4B, C) of glutamate transmission. Furthermore, we also recorded spontaneous GABA transmission. Interestingly, we detected a significant decrease in the frequency of GABA transmission in the cKO mice (Fig. 4D, F). However, the amplitude of GABA transmission was not changed (Fig. 4E, F).

Because we detected GABAergic changes in our electrophysiology data, we further investigated the expression level of genes involved in glutamate and GABA transmission. After surveying several receptor and transporter subtypes, we discovered that the protein level of GABA_A_ receptor β3 subunit was significantly reduced by threefold (Fig. 4G). To determine if the decrease in GABA frequency in our electrophysiology data was caused by deficits in synthesizing GABA, we examined GAD67 protein level (Fig. 4H). GAD67 levels were found to be normal, indicating that GABA synthesis is not the cause of the observed decrease in GABA transmission. Similarly, we also didn’t detect significant changes of protein levels of GRIA1 (glutamate receptor 1), GABBR2 (GABA-B receptor subunit 2), DLG4 (PSD95), GPHN (gephyrin), SLC1A2 (glutamate/aspartate transporter 2), SLC32A1 (vesicular GABA transporter), and SLC12A2 (NKCC1) in the brains of control and KO groups (Supplemental Fig. S6).

### RNAseq analysis of the E12 cortex

**Fig. 5:**
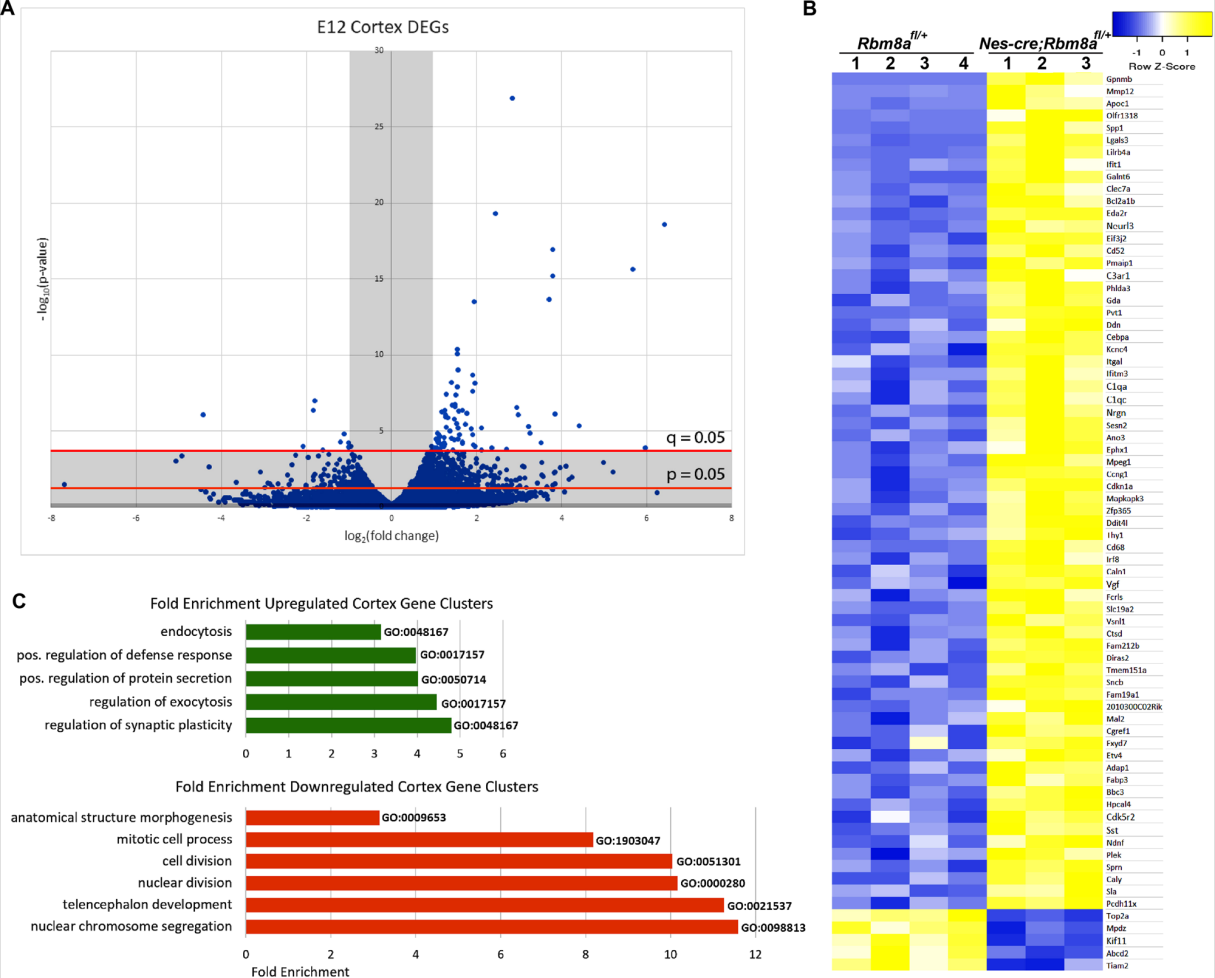
RNAseq analyses of all quantifiable expression changes in the cortex of *Rbm8a* cKO mice at E12. **A** Volcano plot of 28,117 genes’ transcripts detected in the RNAseq data. A total of 1073 genes were expressed differently with *p* **<** 0.05, 430 genes were expressed differently with *p* **<** 0.01, and 87 genes were expressed differently with *q* < 0.05. A total of 84 DEGs with significant *q* values were upregulated or downregulated at least twofold. The *p* and *q* cutoffs are shown. **B** Heat map of RNA transcript readings for all known genes with significant expression changes in the E12 cKO mouse cortex (*q* < 0.05). From the RNAseq data, the transcript counts of each gene were compared between the six mice. Of the 87 DEGs, 14 remain uncharacterized and have been excluded from the figure. **C** Enriched gene clusters of the E12 cortex regulated endocytosis, immune responses, cell secretions, and synaptic plasticity. Depleted gene clusters of the E12 cortex were mainly involved in cell cycle processes. Enriched gene clusters were identified among upregulated and downregulated genes. Selected clusters with more than twofold enrichment are shown with their GO accession numbers to the left of the bars.

To further determine the transcriptomic changes in our cKO mice, we conducted RNAseq using the neocortices at E12 because the Nes-cre line normally expresses cre recombinase at early embryonic stage, around E11. Despite prominent brain abnormalities in the postnatal stage, we believe that these late defects are caused by *Rbm8a* loss-of-function in the embryonic brain. Reads from the Illumina HiSeq 2500 were mappable to ~50,000 known transcripts in the mouse genome. A volcano plot was generated to display all genes that had quantifiable transcript readings in both the WT and cKO cortices (Fig. 5A). This accounts for ~28,000 genes, microRNAs, and long noncoding RNAs, and covers more than half of the referenced transcripts. In the E12 cortex, 430 genes showed altered expression with significant *p* values (*p* < 0.01) and 87 differentially expressed genes (DEGs) showed at least twofold change with significant *q* values (*q* < 0.05). Of these 87 DEGs, 73 had previously identified functions. The original RNA transcript readings for these 73 DEGs are compared between the WT and cKO mice (Fig. 5B).

A total of 19 of the DEGs were identified to be relevant to the central nervous system (CNS), in function and/or expression (Supplemental Table 1). Enrichment analysis was performed using the E12 datasets. The gene pool was expanded to include all DEGs that were significant at *p* < 0.01 because there were not enough DEGs significant at *q* < 0.05 for the software to detect enrichment of any gene clusters. In the E12 cortex, several upregulated gene clusters seemed to revolve around trafficking of cell components, including endocytosis, exocytosis, and protein secretions, which apparently affect synaptic plasticity (Fig. 5C). Downregulated genes generally showed greater enrichment than upregulated genes. Enriched clusters of downregulated genes would correspond to depletions of those gene clusters in the cKO mice. In the cortex, most of the notable enrichments pertained to cell division or differentiation. Enrichment of neuron fate specification (GO:0048665), oligodendrocyte differentiation (GO:0048709), and the Notch signaling pathway (GO: 0007219) clusters (not shown) would affect differentiation of progenitor cells. In addition, genes for the positive regulation of neural precursors (GO:2000179) were depleted (Fig. 5C). In summary, in the E12 mouse cortex, we observed changes in gene expression relevant to myelination, DNA replication, and neurodevelopment.

### *Rbm8a* haploinsufficiency in NKX2.1+ progenitors leads to developmental deficit in PV interneuron

**Fig. 6:**
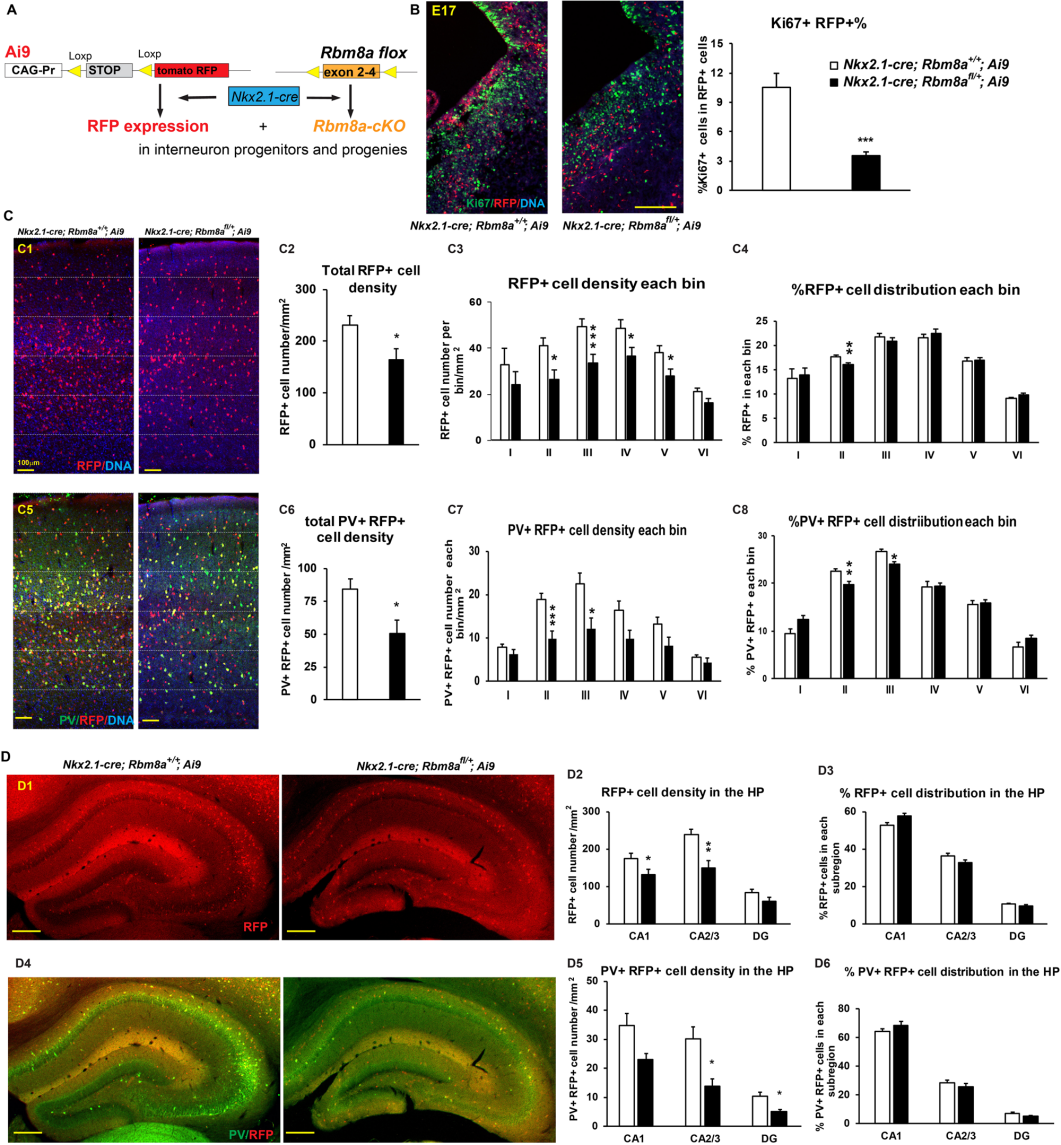
Altered interneuron distribution and number in *Nkx2.1-cre; Rbm8a*^*fl/+*^ mice. **A** Generation of *Nkx2.1-cre; Rbm8a*^*fl/+*^*; Ai9* mouse model. **B** Mice have significantly fewer proliferating RFP+ cells in the GE on E17. Scale bar = 50 µm. Quantification of the percentage of proliferating Ki67+ cells normalizing to total RFP+ cells in the GE (***p < 0.001; *t* test, *n* = 3). **C** Analyses of total RFP+ cells in the DLPFC of *Nkx2.1-cre; Rbm8a*^*+/+*^*; Ai9* and *Nkx2.1-cre; Rbm8a*^*fl/+*^*; Ai9* mice. (C1) Representative figures show RFP+ cells in the DLPFC of *Nkx2.1-cre; Rbm8a*^*+/+*^*; Ai9* and *Nkx2.1-cre; Rbm8a*^*fl/+*^*; Ai9* mice. Scale bar = 50 µm. (C2) Quantification of total RFP+ cell density in the DLPFC of *Nkx2.1-cre; Rbm8a*^*+/+*^*; Ai9* and *Nkx2.1-cre; Rbm8a*^*fl/+*^*; Ai9* mice. (C3) Quantification of RFP+ cell density in each bin of the DLPFC. The bar graph shows the RFP+ cell number in each bin divided by area. (C4) Quantification of percentages of RFP+ cells in each bin of the DLPFC. The bar graph shows the percentage of RFP+ cells in each bin to total RFP+ cells in all bins. (C5) Co-immunostaining of PV interneurons in the section shown in C1. Scale bar = 50 µm. (C6) Quantification of total PV+ and RFP+ cell density in the DLPFC. (C7) Quantification of PV+ RFP+ cell density in each bin of the DLPFC. (C8) Quantification of percentages of PV+ RFP+ cells in each bin of the DLPFC. **D** Analyses of total RFP+ cells in the HP of *Nkx2.1-cre; Rbm8a*^*+/+*^*; Ai9* and *Nkx2.1-cre; Rbm8a*^*fl/+*^*; Ai9* mice. (D1) Representative figures show RFP+ cells in the HP of *Nkx2.1-cre; Rbm8a*^*+/+*^*; Ai9* and *Nkx2.1-cre; Rbm8a*^*fl/+*^*; Ai9* mice. Scale bar = 50 µm. (D2) Quantification of RFP+ cell density in subregions of the HP. The bar graph shows the RFP+ cell number in each subregion divided by area. (D3) Quantification of percentages of RFP+ cells in each subregion of the HP. The bar graph shows the percentage of RFP+ cells in each subregion to total RFP+ cells in the whole HP. (D4) Co-immunostaining of PV interneurons in the section shown in D1. Scale bar = 50 µm. (D5) Quantification of PV+ RFP+ cell density in subregions of the HP. (D6) Quantification of percentages of PV+ RFP+ cells in each subregion of the HP. *p < 0.05; **p < 0.01; ***p < 0.001, *t* test, *n* = 3. Bar graphs represent means ± SEMs.

Since we observed profound deficits in the interneuron distribution in global brain *Rbm8a* KO mice, we further investigated if these changes were due to defective intrinsic cues within the interneurons, or due to abnormal extrinsic cues from the excitatory neurons. To address this question, we crossed our *Rbm8a*^*fl/fl*^ mice with *Nkx2.1-cre* mice to selectively delete *Rbm8a* in interneuron progenitors, but to leave it intact in excitatory neurons and in the rest of the nervous system. Loss of *Rbm8a* in interneurons didn’t significantly change the overall growth, breeding, and life span. The brain size in length and width are normal (Supplemental Fig. S7A, C, D). However, we detected a small but significant reduction in brain weight (Supplemental Fig. S7B), suggesting a subtle defect in brain development. To specifically trace progenies (RFP+ cells) of NKX2.1*+* progenitors, when *Rbm8a* expression is reduced in vivo, we crossed *Nkx2.1-cre; Rbm8a*^*fl/+*^ mice with cre-dependent Ai9 reporter line (Fig. 6A). To examine the requirement of *Rbm8a* in proliferation of interneuron progenitors, we checked the cell cycle marker, Ki67, in RFP+ cells at the GE region of *Nkx2.1-cre; Rbm8a*^*+/+*^*; Ai9* and *Nkx2.1-cre; Rbm8a*^*fl/+*^*; Ai9* brains (Fig. 6B). Percentage of Ki67 and RFP double-positive cells were significantly reduced about twofold (10.5 ± 1.5% in WT versus 3.5 ± 0.4% in cKO mice, *p* = 0.00075), supporting that *Rbm8a* is required for interneuron progenitor proliferation.

To measure the differentiation process of NKX2.1+ progenitors, we first quantified total RFP+ cells in the DLPFC at P17, the same time we measured in *Nes*-*cre; Rbm8a*^*fl/+*^ line (Fig. 6C1–C4). Consistent with decreased progenitor proliferation in the GE region (Fig. 6B), total RFP+ cell density was significantly reduced in the DLPFC of *Nkx2.1-cre; Rbm8a*^*fl/+*^ mice (Fig. 6C2). As in Fig. 3, we further divided the DLPFC into six bins and quantified RFP+ neuron distribution in each bin. The RFP+ cell numbers were significantly decreased in the bins II–V (Fig. 6C3). When we calculated the percentage of RFP+ cells in each bin, bin II still showed a significant difference (Fig. 6C4, *p* < 0.01), suggesting that *Rbm8a* haploinsufficiency in interneuron progenitors not only affects proliferation, but also migration.

As NKX2.1+ progenitors can give rise to PV interneurons^21^, we stained *Nkx2.1-cre; Rbm8a*^*+/+*^*; Ai9* and *Nkx2.1-cre; Rbm8a*^*fl/+*^*; Ai9* brain sections with PV antibody, and quantified PV+ RFP+ cells in the DLPFC region (Fig. 6C5–C8). Consistently, total PV+ RFP+ cells were also significantly reduced in *Nkx2.1-cre; Rbm8a*^*fl/+*^*; Ai9* mice (84 ± 7.8/mm^2^ in control versus 50.6 ± 9.9/mm^2^ in cKO, *p* < 0.05; Fig. 6C6). After dividing into bins, significant changes were detected in bins II and III for both density and percentage (Fig. 6C7, C8). We further analyze pure PV+ neurons in the DLPFC, and detected a defective density in bin III (Supplemental Fig. S8A) and a distribution change in bin I (Supplemental Fig. S8B). Because NKX2.1+ progenitors can produce other types of interneurons, we further checked the population of PV negative RFP+ cells (PV− RFP+). However, both the cell density and percentage of distribution of PV− RFP+ cells were not altered (Supplemental Fig. S8C, D). These data together support that *Rbm8a* deficiency in NKX2.1+ progenitors play a major role in the differentiation of PV+ interneurons, but with minor effects on other progenies derived from NKX2.1+ progenitors.

To test if *Rbm8a* deficiency also causes interneuron defects in the HP, we measured similar parameters in the subregions of the HP (Fig. 6D1–D6). Interestingly, we observed ~40% reduction of RFP+ cell density in the CA2/3 region (*p* < 0.005, Fig. 6D2), yet the percentage of RFP+ cells in each subregion was not changed (Fig. 6D3). Consistently, PV+ RFP+ neuron density was significantly decreased in the CA2/3 and the DG regions in *Nkx2.1-cre; Rbm8a*^*fl/+*^*; Ai9* mice (Fig. 6D5), but the percentage of PV+ RFP+ neuron in each subregion of the HP was not changed (Fig. 6D6). When we examined the total PV+ neurons or PV− RFP+ neurons in the HP (Supplemental Fig. S9), consistently, cell density was significantly reduced by 50% in the CA2/3 region (Supplemental Fig. S9A, C), suggesting that intereneurons in the CA2/3 region are sensitive for *Rbm8a* deficiency. However, the overall percentage of cell distribution in each region was not altered (Supplemental Figure S9B, D). Therefore, we concluded that *Rbm8a* haploinsufficiency does change intrinsic cues within the interneuron progenitors.

## Discussion

The development of the nervous system involves the accurate NP proliferation, differentiation, and neuronal migration. When this process is perturbed, it can result in abnormal ratios of cellular subtypes, deficiencies in cell number, malformed neural structures, and cellular architecture, and eventually developmental disorders. Previously, we have demonstrated that *Rbm8a* is a positive regulator of NP proliferation^17^. Using *in utero* electroporation, we have shown that KD of *Rbm8a* leads to increased cell number in the CP and fewer cell number in the VZ/SVZ, while overexpression leads to a decreased cell number in the CP and increased cell number in the VZ/SVZ^17^. Here, we illustrate *Rbm8a’s* pivotal role in the differentiation and migration of both cortical NPs and interneuron NPs. *Rbm8a* cKO in neural stem cells results in early postnatal lethality, microcephaly, and leads to decreased number of PV+ and NPY+ cortical interneurons, abnormal distribution of PV+, SST+, and NPY+ interneurons in the cortex, and aberrant GABA transmission. Consistently, *Rbm8a* haploinsufficiency in NKX2.1+ interneuron progenitors causes a significant reduction of progenitor proliferation and abnormal distribution of interneuron progenies, especially PV+ interneurons. The RNAseq results reveal some important neurodevelopmental genes associated with *Rbm8a-*mediated defects. Thus, our results demonstrate that *Rbm8a* is essential for the neurodevelopment, and loss of even one copy of this gene can lead to the severe phenotypes, including microcephaly, a disruption in the excitatory/inhibitory (E/I) balance in the brain and early postnatal lethality.

Our study demonstrates that *Rbm8a* is involved in the differentiation of interneuron subtypes. During the process of the interneuron development, the absence of *Rbm8a* leads to changes in cell number, migration, and size of interneuron subtypes. The novel role of *Rbm8a* in interneuron fate specification and migration could arise, as a result of abnormal tangential migration of interneuron progenitors, combined with juxtacrine signaling of excitatory neurons. Nestin promoter driven-cre is expressed on E11, which would lead to recombination in early embryonic development in the *Nes-cre*; *Rbm8a*^*fl/+*^mice. *Rbm8a* has been implicated in the control of proliferation, differentiation, and migration of excitatory NPs in the VZ/SVZ^17,18^. The current study investigates the differentiation of cells into various interneuron subtypes in the *Nes-cre*; *Rbm8a*^*fl/+*^mice, which arise from the progenitor pool in the GE. To examine whether changes in interneuron progenitors are responsible for this phenotype, we injected pregnant dams with EdU 1 h before perfusion, to label cells in the S phase of the cell cycle and stained brain section with cell cycle marker, Ki67. Significant decreases in the numbers of EdU+ and Ki67+ cells were detected in the GE of *Nes-cre*; *Rbm8a*^*fl/+*^mice, illustrating that deficits in proliferation are partially responsible for the observed phenotype. Interestingly, expression levels of several key transcriptional factors for interneuron development, such as SOX6^22^ and ARX^23^ have been significantly downregulated, but DLX1, DLX2, and NKX2.1 were unchanged (Fig. 2D). Although we did not detect changes in the number of NKX2.1+ progenitors in the GE, the cell distribution was altered (Supplemental Fig. S4). Thus, *Rbm8a* deficiency could affect both excitatory and inhibitory neuron development.

Consistent with a decrease in the proliferation of general interneuron progenitors, our data show a significant decrease in the number of PV+ interneurons in the cortex of *Nes-cre; Rbm8a*^*fl/+*^ mice (Fig. 3A, B). As PV+ interneurons are the most populous interneuron subtype in the cortex, this deficit has the ability to significantly alter the E/I balance. In addition, the distribution of PV+ interneurons in the cortex is altered, with more cells residing in the superficial layers, at the expense of the deeper layers. A disruption in the cell numbers in each cortical layer, especially inhibitory cells, can seriously disrupt the neuronal networks of the brain, which can negatively affect behavior, cognition, and even autonomous function^24,25^. In fact, abnormal PV and GAD67, two proteins expressed in interneurons, are observed in human patients with SZ, and mouse models of autism often show reduced number of PV+ interneurons^9,26–29^. In addition, changes of the brain size in early development are characteristic of ASDs. Both microcephaly and macrocephaly during early development have been observed in humans with ASD^30^.

Abnormal brain size could also affect the cell size in the brain. Neuron size is highly regulated during development to enable the proper connectivity of neuronal circuits in the adult, which is critical to normal brain function. Aberrations in size and morphology have been shown to be associated with or linked to various brain disorders^31,32^. We detected increased soma sizes of interneurons in in the cortex of *Nes-cre; Rbm8a*^*fl/+*^ mice. Changes in cell size would alter the surface area to increase or decrease membrane bound proteins, such receptors and transporters, thereby regulating neural signal transmission and connectivity. Consistently, we observed upregulation of multiple surface receptors and membrane proteins, such as ectodysplasin A2 receptor (*Eda2r*), complement C3a receptor 1 (*C3ar1*), and transmembrane protein 151 A (*Trem151a*). Multiple signaling pathways, including PI3K/PTEN/ mTOR cascade^33,34^ and mechanical signals^35^, are important for regulating cell size. Particularly, integrins can link extracellular mechanical signals with mTOR signaling^36^. Interestingly, our RNAseq revealed that *Itgal* encoding integrin α L chain, is upregulated over threefold. It would be interesting to follow up if integrin is responsible for cell size phenotype in the future study. In contrast, the soma sizes of excitatory neurons in the layer 2 of the cortex show no significant difference in *Rbm8a* KO brains (data not shown). Thus, cell size phenotype in *Rbm8a* KO mice could be cell type specific.

We hypothesized that the electrophysiological properties of the pyramidal cells in the cortex were likely altered. To assess this, we conducted spontaneous transmitter recordings in the somatosensory cortex, and discovered a significant decrease in the frequency of GABA transmission, but no change in the frequency of glutamate transmission, or the amplitude of GABA or glutamate transmission. Paired with our data indicating decreased interneuron number, we speculate this decrease in the frequency of GABA transmission is due to the decreased number of interneurons, and not due to the neurotransmitter volume in the synaptic vesicles. This hypothesis is further supported by a significant reduction of GABA-A receptor β3 subunit, but no change in GAD67 expression, which indicates no deficiency in synthesizing GABA (Fig. 5). The decrease in cell number was not observed with SST+ cells, but was with NPY+ cells. The abnormal laminar distribution was observed for all interneuron cell types. Taken together, this illustrated a profound deficit in inhibitory circuitry in the *Nes-cre; Rbm8a*^*fl/+*^ mice.

It is possible that the profound deficits in interneuron patterning in the cortex of *Nes-cre; Rbm8a*^*fl/+*^ mice are due to aberrant juxtacrine signals from the already perturbed excitatory neurons. To investigate this, we crossed our *Rbm8a*^*fl/fl*^ mice with and *Nkx2.1-cre* and *Ai9* reporter line. *Nkx2.1-cre; Rbm8a*^*fl/+*^ mice will only have *Rbm8a* heterozygous deletion in interneuron progenitors. This will allow us to assess whether our interneuron phenotype is due to intrinsic changes in the interneuron progenitors (cell autonomous) or due to extrinsic cues from the already perturbed excitatory interneurons (cell nonautonomous). By looking at the embryonic and postnatal distribution of the RFP+ progenies in these mice, we will also be able to tell if the migration of these progenitors is affected. Consistent with decreased progenitor proliferation in the GE of *Nes-cre* cKO mice, the percentage of RFP+ progenitors expressing cell cycle marker Ki67 was significantly reduced at E17 and their progenies were decreased in the postnatal *Nkx2.1-cre; Rbm8a*^*fl/+*^ mice (Fig. 6). Moreover, migration of RFP+ interneurons into the DLPFC and HP was defective. Interestingly, PV+ interneurons contribute to the majority of defects rather than other types of interneurons. This indicates that *Rbm8a* deficiency can modulate interneuron differentiation through intrinsic, cell autonomous mechanisms.

NMD is an RNA surveillance mechanism that controls RNA stability, and ensures the speedy degradation of erroneous and unnecessary transcripts. This mechanism depends on several core factors in the EJC, eIF4A3, RBM8A, MAGOH, and CASC3, as well as peripheral factors to distinguish PTCs from normal stop codons in transcripts. Recently, emerging evidence has indicated that NMD factors are associated with neurodevelopmental disorders, such as ASD and ID^12,37^. Our mouse phenotype is consistent with genetic studies on humans, which have associated RBM8A with neurodevelopmental disorders, such as microcephaly. Specifically, compound mutations in *RBM8A* lead to TAR syndrome, a disorder characterized by missing radius bones, low blood platelet counts, and in 7% of cases ID^16^. In addition, *RBM8A* is located in the 1q21 region of the genome, which is associated with neurodevelopmental disorders, such as autism and SZ^38,39^. These studies indicate a potential role for RBM8A in the pathophysiology of neurodevelopmental disorders. In addition, overexpression of RBM8A in the DG of mice has been associated with abnormal behaviors, such as increased anxiety, and abnormal social behaviors^19^ and dysregulation of RBM8A has also been implicated in cancer and Alzheimer’s disease^40,41^. These behaviors are reminiscent of the behaviors seen in patients with neurodevelopmental disorders, and further suggests *RBM8A’s* role in these disorders^42^. As mentioned previously, RBM8A is a member of the EJC, along with MAGOH, eIF4A3, and CASC3. Interestingly, *Magoh* cKO leads to similar phenotypes as *Rbm8a*, namely microcephaly, abnormal asymmetric division and interneuron differentiation^43–45^. These genetic indications of RBM8A’s role in psychiatric disease are especially interesting in light of the neurogenesis and cell migration deficits associated with *Nes-cre; RBM8a*^*fl/+*^ mice. In fact, studies have revealed that children with ASDs have disorganized cortices, as seen using postmortem immunohistological analyses. Thus, our study on the *Rbm8a* cKO mice may further reveal its potential role contributing to the pathogenesis of neurological diseases.

To determine the molecular changes downstream of *Rbm8a*, our RNAseq analysis revealed multiple DEGs. The two downregulated genes among these were *Abcd2* and *Top2a*. *Abcd2* is a membrane transporter that is involved in production and/or maintenance of the myelin sheath^46^, and *Top2a* is a topoisomerase, a protein that directly participates in DNA replication, specifically expressed in the CNS^47^. Meanwhile, another gene involved in myelin maintenance, *Mal2*, is upregulated more than twofold. Two CNS-related DEGs were *Kcnc4*, a voltage-gated potassium ion channel^48^, and *Gda*, a guanine deaminase concentrated in the brain that regulates microtubule assembly^49^. *Kcnc4* conducts potassium outward following an action potential, restoring the membrane to resting state^48^. Likewise, the increase in *Gda* expression could indicate a misregulation of the cytoskeleton or an increase in cellular outgrowths. Among the other upregulated, CNS-related DEGs, *Hpcal4*, *Caln1*, and *Nrgn* are involved in calcium-dependent signaling pathways in the neuronal cytoplasm. *Hpcal4* and *Caln1* directly bind Ca^2+^ (ref. ^50,51^), potentially acting as calcium sensors in the presynaptic terminal. *Nrgn* binds calmodulin at synapses and may participate in long-term potentiation^52^. This suggests that *Rbm8a* might influence both immediate synaptic transmission and long-term synaptic excitability by regulating genes involved in calcium signaling. Interestingly, we detected more upregulated genes than downregulated genes. Increased RNA stability caused by defective NMD could explain upregulation of some hits. For example, *Gpnmb*, *Mmp12*, and *Lilrb4a*, express alternatively spliced transcripts *Gpnmb-202* (ENSMUST00000203757.1), *Mmp12-204* (ENSMUST00000127722.1), and *lilrb4a-205* (ENSMUST00000218617.1) that normally are undergone NMD, but could be stabilized in *Rbm8a* cKO brain. However, these genes cannot explain all upregulations observed in *Rbm8a* cKO mice. We believe that some genes may be upregulated indirectly from other gene changes. Future studies are necessary to examine how NMD and EJCs are coordinated in specific neuronal types to regulate transcript abundance, and to contribute to normal development and pathophysiology in vivo.

## Materials and methods

### Mice

All procedures on mice were reviewed and approved by the Pennsylvania State University IACUC committee, under IACUC protocol number 44057. Wild-type male and female C57BL/6 N mice were obtained from Taconinc. Mice were hosted by sex (2–5 mice per cage) in a room with a light/dark cycle at 12 h intervals, and provided *ad libitum* access to food and water. The cKO strategy was designed using the cre-loxp system of PGKneolox2DTA vector^53^. To make the targeting construct, a 4.5 kb 5′ homologous region to the exon 2 was subcloned into the SacII and NotI sites in the vector. Loxp sites flanked exons 2–4, and a neomycin-resistance cassette (PGK-neo) was at the downstream of exon 4. PGK-neo is flanked by flpe recombinase recognition sites (FRT). A 2.7 kb 3′ homologous region was subcloned at NheI and EcoRV sites of the vector. To generate *Rbm8a* cKO mice, the linearized targeting construct was electroporated into embryonic stem (ES) cells derived from C57BL/6J/129S6 hybrid line. Two targeted ES clones were identified from 196 G418-resistant clones. The positive clones were identified by long-range PCR using 5’ primer pair (ACCTGGGTAATTTAGCAAGACT and ACCTGGGTAATTTAGCAAGACT) to examine 5’ recombination and 3’ primer pairs (TCTTCTGAGGGGATCAATTCTC and GCCTGTAGCAGCAATAGCCT). Correct ES cells were injected into blastocysts of C57BL/6J mice at the Transgenic Core of University of Rochester Medical School, and the chimeras were crossed with C57BL/6J mice to obtain germ line transmission. The resulting progeny was then crossed with Actin-Flipase mice to remove the Neo cassette (used for selection). The mice were then backcrossed to create homozygous floxed mice without the Neo cassette. These mice were then crossed with *Nes-Cre* (Jackson lab # 003771, *Tg(Nes-cre)1Kln/J*), and *Nkx2.1-cre* (Jackson lab # 8661, *Tg (Nkx2.1 cre)2Sand*) lines, to selectively KO *Rbm8a* in neural stem cells and interneuron progenitors. *Ai9* reporter line was obtained from Jackson lab (Jackson lab # 7909).

### Immunocytochemistry/immunohistochemistry

Mice at P17 were anesthetized with 2.5% avertin and then perfused with ACSF followed by 4% paraformalydehyde (PFA)/PBS. Brains were then postfixed for a minimum of 24 h in 4% PFA in 4 °C. The brains were cut into 70 micron slices using a vibratome. Slices were blocked and permeablized using 5% donkey serum in PBST (0.3% triton). Primary antibodies used consisted of rabbit anti-PV (Abcam ab11427), rat anti-SST (Millipore, MAB354), and rabbit anti-NPY (Abcam, ab180809) antibodies, in 1/200 dilutions. Slices were incubated at room temperature (RT) in primary antibody overnight with rocking. Secondary antibodies included donkey anti-rabbit cy3 (Jackson ImmunoResearch), donkey anti-rat cy3 (Jackson ImmunoResearch), RedDot (Biotium), or DAPI (Sigma). Secondary antibodies were used at a 1:250 dilutions, and slices were rocked in secondary antibodies for 1 h at RT. During the first PBS wash, DAPI was added to the PBS solution.

### Organ measurements

Organs were dissected out immediately after sacrifice, and the excess liquid was dabbed onto a paper towel. The weigh boat was tared and the organ was placed on the weigh boat to determine the weight in grams. The organ weight was normalized to the total weight of the mouse to determine the percentage of body weight that the organ occupied. Three mice of each genotype were assessed and a Student’s *t* test was used to determine the statistical significance.

### Western blot

Immunoblots were performed as previously described^17^. The total proteins were prepared from whole cell extracts from whole mouse brains at postnatal day 17 using 500 µl RIPA cell lysis buffer. RIPA cell lysis buffer includes 10 mM Tris-HCl (pH 8.0), 140 mM NaCl, 1 mM EDTA, 1% Triton X-100, 0.1% SDS, 1 mM β-glycerophosphate, 1 mM Na_3_VO_4_, 2 µg/ml aprotinin, 1 mM PMSF, and 130 µM bestatin. The tissue was homogenized on ice for 20 min, the supernatant from each sample was collected and stored in aliquots at −80 °C. For each sample, the protein concentration was determined by Bradford assay (ThermoFisher Scientific). A total of 20–50 µg of cell lysate were resolved by 8–12% SDS–polyacrylamide gel electrophoresis, and were transferred to nitrocellulose membranes. The blots were blocked with 5% milk in TBST (10 mM Tric-HCl, pH 8.0, 150 mM NaCl, and 0.5% Triton X-100) for 1 h at RT. Blots were then incubated with mouse anti-RBM8a, or mouse anti-actin antibody in 5% milk in TBST overnight at 4 °C, with shaking. The secondary antibody was a donkey anti-mouse IgG (LI-COR). Immunoreactivity was detected using the LI-COR Odyssey imaging system according to the company’s instructions.

### Image acquisition, statistical analysis, and quantification

Images used for quantification were acquired using a Zeiss LSM Image browser and ImageJ (version 1.5). Images shown in the publication were acquired using Zeiss Pascal confocal, a Zeiss Axio Observer with ApoTome.2 LSM 880. A Student’s *t* test was used to perform statistical analysis. All bar graphs were plotted as mean ± standard error of the mean (SEM). In all analyses, *p* < 0.05 was considered statistically significant. DAPI quantification was conducted using the ITCN plugin on ImageJ. All immunohistological quantification and analysis were done blindly based on 3–4 control and cKO mice. 3–10 brain slices were used from each mouse. The analysis of the distribution of the interneurons of the cortex was conducted by dividing the cortex into six equal bins^54^. Cell number analyses (200–1000 cells/section) were done by dividing the number of interneurons in the cortex by the number of DAPI positive cells or total area (mm^2^). For the cell size analysis, an additional test of Cohens *δ* was performed to determine the effect size (since we sampled over 1000 cells).

### Slice electrophysiology

Whole-cell patch-clamp electrophysiology was conducted as published previously (Crowley et al., 2016; Pleil et al., 2015). Briefly, mice were anesthetized with 1–2% isoflurane anesthesia and rapidly decapitated. Brains were removed and immediately placed in ice-cold oxygenated (95% O_2_/5% CO_2_) high-sucrose artificial cerebrospinal fluid (aCSF) (containing the following, in mM: 194 sucrose, 20 NaCl, 4.4 KCl, 2 CaCl_2_, 1 MgCl_2_, 1.2 NaH_2_PO_4_, 10.0 glucose, and 26.0 NaHCO_3_). A total of 300 mM cortical slices containing the somatosensory cortex were prepared on a Leica VT1200s vibratome, and immediately placed in oxygenated heated (30 °C) aCSF (124 mM NaCl, 4.4 mM KCl, 2 mM CaCl, 1.2 mM MgSO_4_, 1 mM NaPO_4_, 10 mM glucose, and 26 mM NaHCO_3_) where they were allowed to recover for at least 1 h. Recording electrodes (3–7 MΩ) were pulled from thin-walled borosilicate glass capillaries with a Flaming-Brown Micropipette Puller. Pyramidal neurons were selected based on morphology and general location. Spontaneous excitatory postsynaptic currents (sEPSCs) and inhibitory postsynaptic currents (sIPSCs) were recorded in the same neurons at a holding potential of −55 mV (sEPSCs) and +10 mV (sIPSCs), using a cesium methansulfonate-based intracellular solution (135 cesium methansulfonate, 10 mM KCl, 1 mM MgCl_2_, 0.2 mM EGTA, 2 mM QX-314, 4 mM MgATP, 0.3 mM GTP, 20 mM phosphocreatine, pH 7.3, and 285–290 mM mOsmol). Signals were digitized at 10 kHz and filtered at 6 kHz using a Multiclamp 700B amplifier, and analyzed using Clampfit 10.3 software (Molecular Devices, Sunnyvale, CA).

### Analysis of RNA-sequencing

Sample preparation for RNA-sequencing was done by seven mouse embryos (four control and three cKO) at E12 were collected for RNA-sequencing. Four of them were *Rbm8a*^*fl/+*^, and three of them were *Nes-cre; Rbm8a*^*fl/+*^. The cortex regions were dissected from the rest of the brain and stored separately. These brain samples were sent to the Penn State Genomics Core Facility for sequencing with the Illumina HiSeq 2500 on a paired-read protocol. Twenty million paired reads were run per sample, producing 40 million total reads per sample.

The raw Illumina data were processed using the featureCounts htseq pipeline^55^, and reads were mapped to the NCBI *Mus musculus* genome, assembly GRCm38.p6. An R-based package called edgeR was used to process and analyze the RNAseq readings^56^. EdgeR uses the exact test for the negative binomial distribution to calculate differential gene expression between two genotypes. Gene dispersion is normalized using the qCML method^57,58^.

DEGs were ranked by their *p* values and adjusted *p* values (*q* values). We identified genes that were significant at *q* < 0.05 in both conditions being compared, and noted whether these shared DEGs had changed in the same direction. The CNS-related DEGs of the E12 cortex were categorized manually, based on literature reports of their known functions and expression patterns.

Overrepresented gene clusters and pathways were identified using the Gene Ontology Consortium’s free online resource, GO enrichment analysis^59^. The software requires an input list with a sufficient number of genes to accurately identify gene cluster enrichments; we began by inputting the DEGs significant at *q* < 0.05, and if this was not sufficient, expanded the input to include DEGs significant at *p* < 0.01. The E12 cortex datasets were analyzed individually, with inputted DEGs further separated by direction of change (upregulation or downregulation). The PANTHER overrepresentation test was used to recognize groups of genes within the DEGs that occurred at significantly higher or lower counts than expected, relative to all known expression patterns in the mouse genome. All RNAseq raw data have been submitted to NCBI (Bioproject PRJNA631303).

### Statistical analysis

Data were analyzed using Excel and SPSS software, and are expressed as means ± SEM. Significances between the experimental group and control group were analyzed by Student’s *t* test and ANOVA. The normal distribution of variables was verified by D’Agostino and Pearson normality tests. In all analyses, *p* < 0.05 was considered statistically significant. The sample numbers were biological replicates as indicated in each figure legend.

## Supplementary information

SUPPLEMENTAL INFORMATION
