# Supplementary material for: Full function of exon junction complex factor, Rbm8a, is critical for interneuron development
Source: Transl Psychiatry. 2020 Nov 5;10:379. doi: 10.1038/s41398-020-01065-0 (PMC7644723; doi:10.1038/s41398-020-01065-0)
Supplement: Supplementary file 1 — SUPPLEMENTAL INFORMATION [file 41398_2020_1065_MOESM1_ESM.docx]

**Supplemental Information**

**Full function of** **exon junction complex factor, *Rbm8a*, is critical for interneuron development**

Colleen McSweeney, Fengping Dong, Miranda Chen, Jessica Vitale, Nicole Crowley, Bernhard Luscher, Donghua Zou, Yingwei Mao

## Supplemental Materials

### Antibodies used in this study

Material Table - Primary antibodies used in this study

| Antibody | Species | Dilution | | Vendor (Catalog #) |
| --- | --- | --- | --- | --- |
|  |  | IHC | WB |  |
| Ki67 | Rabbit | 1/100 |  | Lab vision (RB90431P1) |
| β-actin | Mouse |  | 1/4000 | Santa Cruz (sc-81178) |
| RBM8A | Mouse |  | 1/500 | Santa Cruz (sc-32312) |
| RFP | Rabbit | 1/250 |  | Genscript (A00682-40) |
| CDK5 | Rabbit |  | 1/500 | Santa Cruz (sc-750) |
| DCX | Goat | 1/250 |  | Santa Cruz (sc-8066) |
| NeuN | Rabbit | 1/100 |  | Millipore (ABN78) |
| GFAP | Mouse | 1/250 |  | 540N1A1 |
| PV | Rabbit | 1/250 |  | Abcam ab11427 |
| SST | Rat | 1/250 |  | Millipore MAB354 |
| NPY | Rabbit | 1/250 |  | Abcam ab180809 |
| NKX2.1 | Rabbit | 1/250 |  | Santa Cruz sc-13040 |
| CC3 | Rabbit | 1/500 |  | Cell Signaling 96615 |
| PH3 | Rabbit | 1/100 |  | Millipore 06-570 |
| CUX1 | Rabbit | 1/300 |  | Santa Cruz sc-13024 |
| DLX1 | mouse |  | 1/200 | Santa Cruz sc-81959 |
| DLX2 | mouse |  | 1/200 | Santa Cruz sc-393879 |
| SOX6 | mouse |  | 1/200 | Santa Cruz sc-393314 |
| ARX | mouse |  | 1/200 | Santa Cruz sc-293449 |
| FMR1 | mouse |  | 1/200 | DHSB 2F5-1 |
| FXR2 | mouse |  | 1/200 | BD 611330 |
| GABA_A_ R β3 | mouse |  | 1/200 | NeuroMab 73-149 |
| GRIA1 | mouse |  | 1/200 | NeuroMab 75-327 |
| GABBR2 | mouse |  | 1/200 | NeuroMab 75-125 |
| GPHN (Gephyrin) | mouse |  | 1/200 | NeuroMab 73-443 |
| DLG4 (PSD95) | mouse |  | 1/200 | NeuroMab 75-028 |
| SLC1A2 (GLT1) | mouse |  | 1/200 | NeuroMab 75-094 |
| SLC32A1 (VGAT) | mouse |  | 1/200 | NeuroMab 75-457 |
| SLC12A2 (NKCC1) | mouse |  | 1/200 | NeuroMab 75-229 |
| GAD67 | chick |  | 1/200 | AVES GAD |

**Supplemental Figures**

**
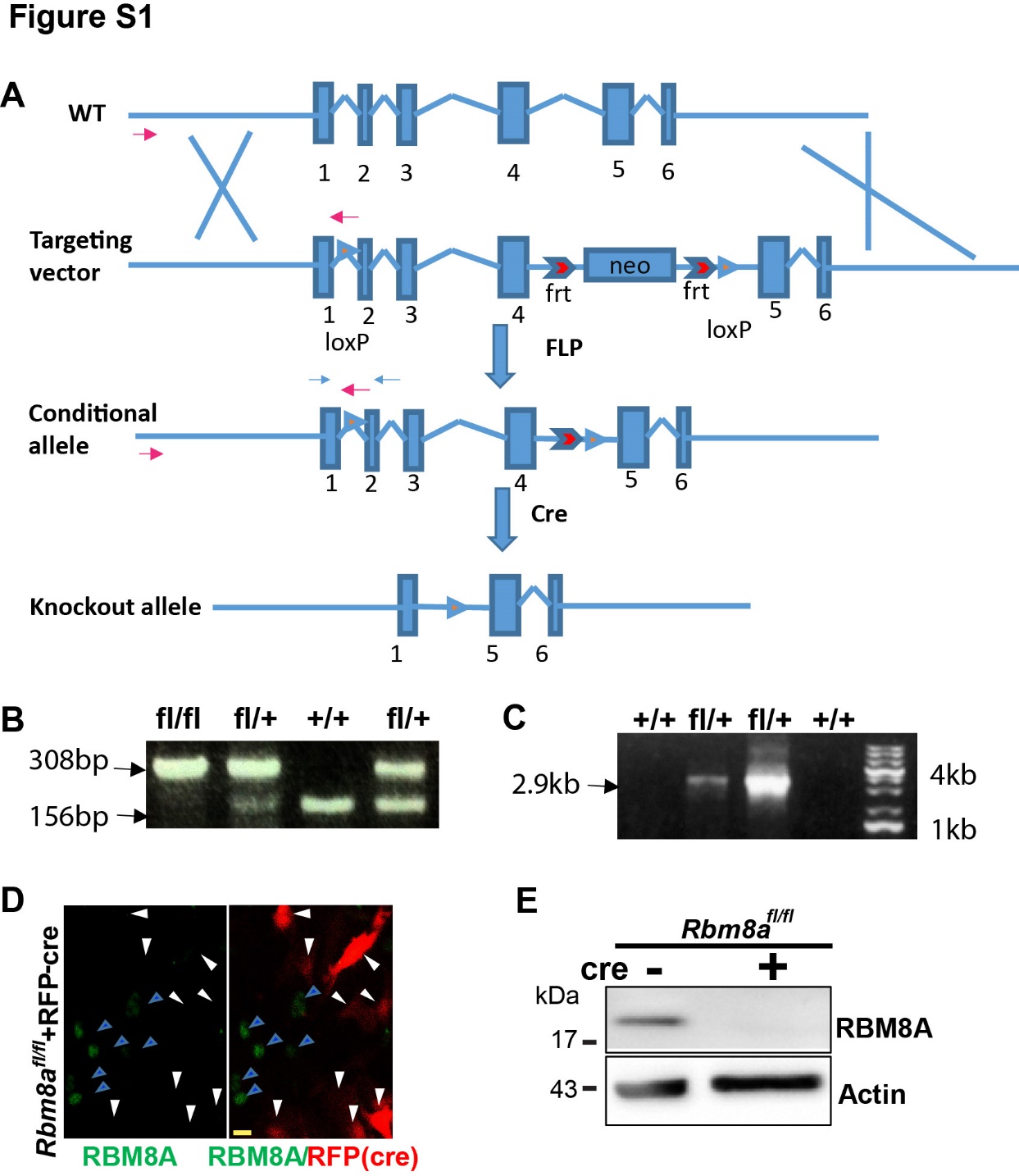
**

S1. Generation of *Rbm8a* cKO mice. (A) Schematic illustrating our knockout strategy for the mice. (B) Genomic DNA was isolated from the selected mice and genotyped for the presence of the loxp/frt site. (C) Genotyping results from long range PCR determined that the homology recombination occured in the correct region of the genome. (D) Primary fibroblasts isolated from *Rbm8a^fl/fl^* mice were infected with RFP-cre virus and stained with RBM8A antibody to demonstrate that RBM8A was removed in the presence of cre. White arrowheads indicate cells expressing cre and blue arrowheads indicate cells without cre. Scale bar=10µm. (E) Immunoblot from lysate obtained from the fibroblast cells with and without cre expression illustrates that RBM8A protein is absent in the presence of cre.


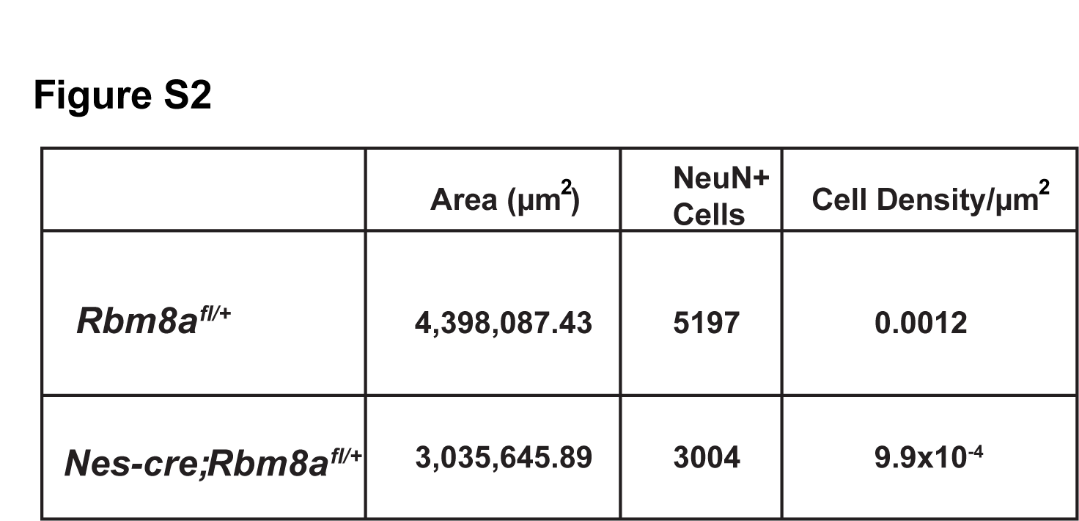


S2. Quantification of the cell density of NeuN+ cells.


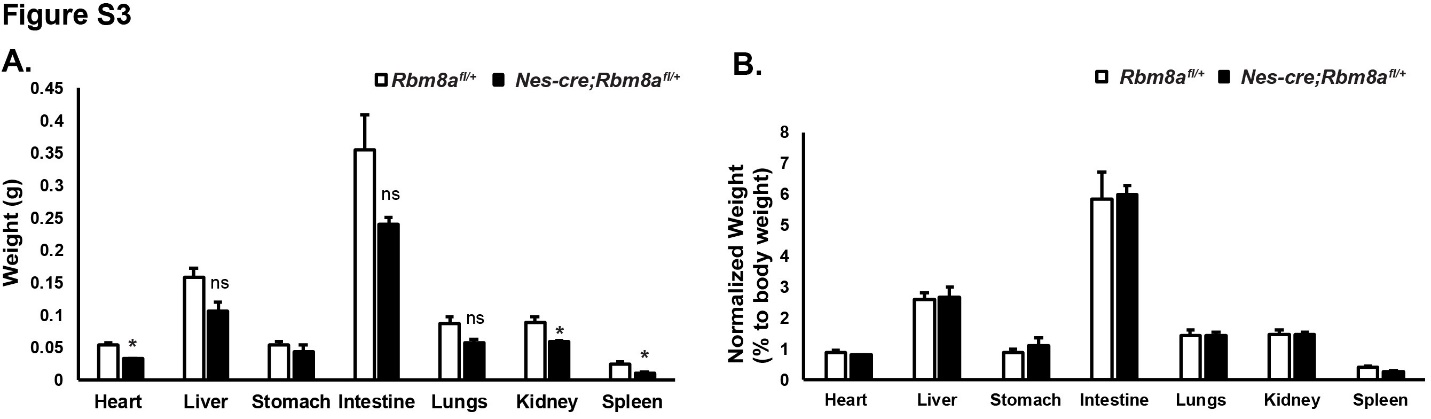


S3. Comparison of weight of organs in *Rbm8a* cKO mice. (A) Weight of organs of the *Nes-cre; Rbm8a^fl/+^* mice compared to littermate controls. (B) Weight of organs was normalized to body weight of the *Nes-cre; Rbm8a^fl/+^* mice (the percentage of the mouse’s body weight of each organ) compared to littermate controls, *p*< 0.05, n=3, Students t-test. ns, not significant.


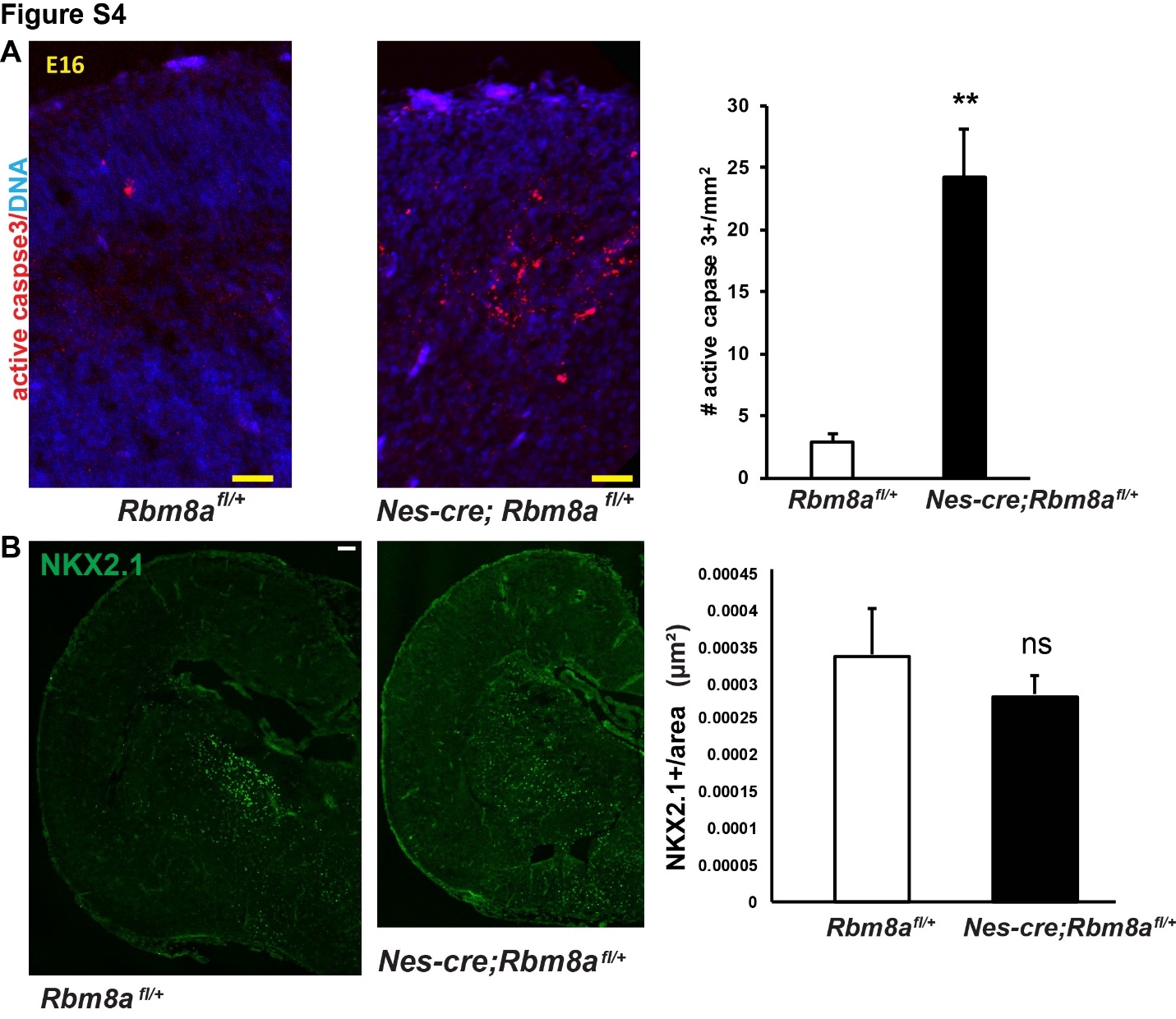


S4. (A) Apoptosis was increased in *Nes-cre; Rbm8a^fl/+^* mice. Active caspase 3 staining is done on E16 *Rbm8a* cKO mouse brains. **, *p*< 0.01, n=4, Students t-test. (B). *Rbm8a* haploinsufficiency does not significantly affect the number of NKX2.1 positive progenitor. The cell density was determined by dividing the number of NKX2.1+ cells by the area of the GE, *p*=0.43, n=3, Students t-test. ns, not significant.


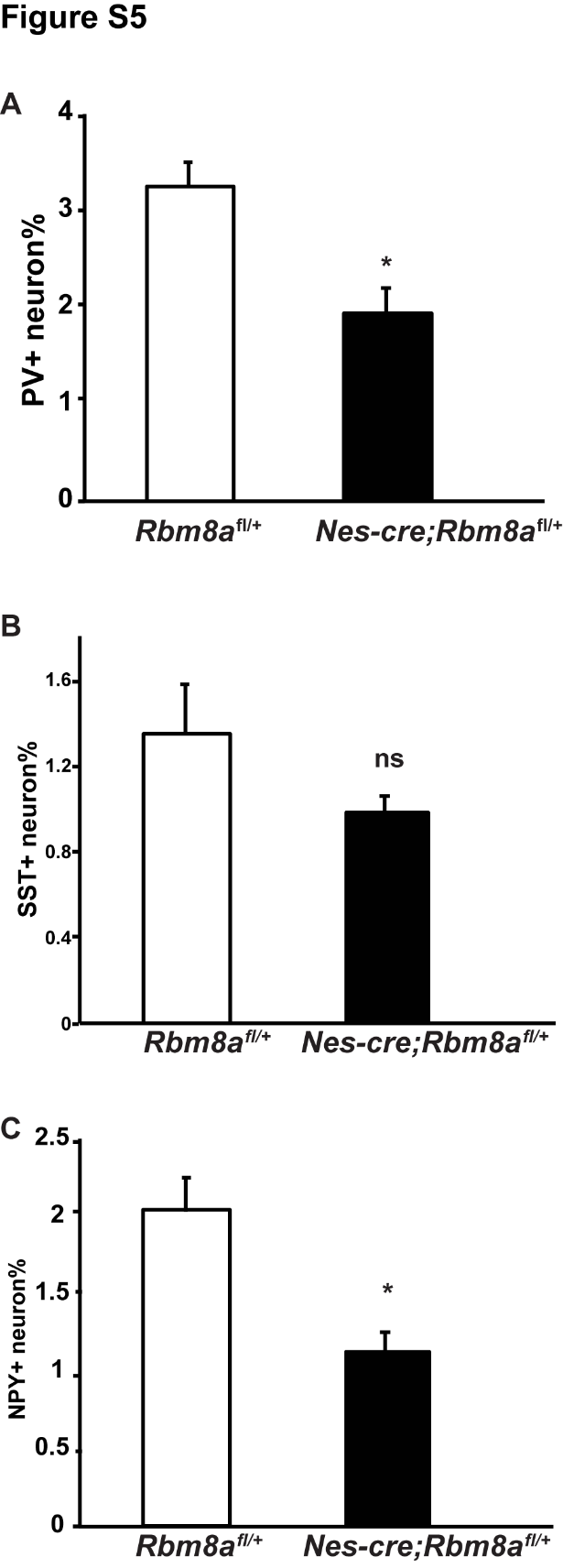


S5. (A) The percentage of PV+ cells in the cortex is significantly less in *Nes-cre; Rbm8a^fl/+^* mice (* *p*<0.05; t test, n=3). (B) The percentage of SST+ cells in the cortex is unchanged in *Nes-cre; Rbm8a^fl/+^* mice. ns, not significant. (C) The percentage of NPY+ cells in the cortex is significantly less in *Nes-cre; Rbm8a^fl/+^* mice (* *p*<0.05; t test, n=3).


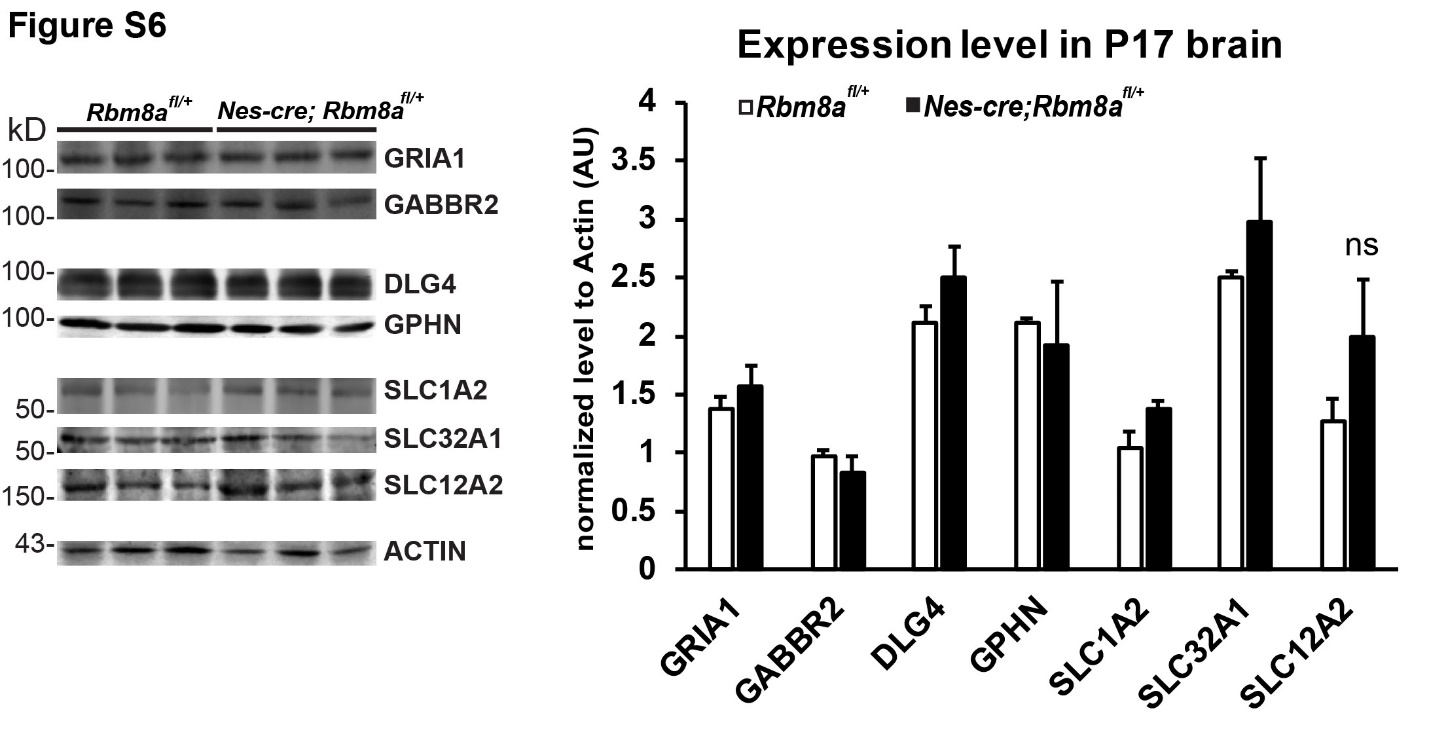


S6. Western blot showed no significant changes of protein expression of GRIA1, GABBR2, DLG4, GPHN, SLC1A2, SLC32A1 and SLC12A2 in *Nes-cre; Rbm8a^fl/+^* brain. n=3, Students t-test.


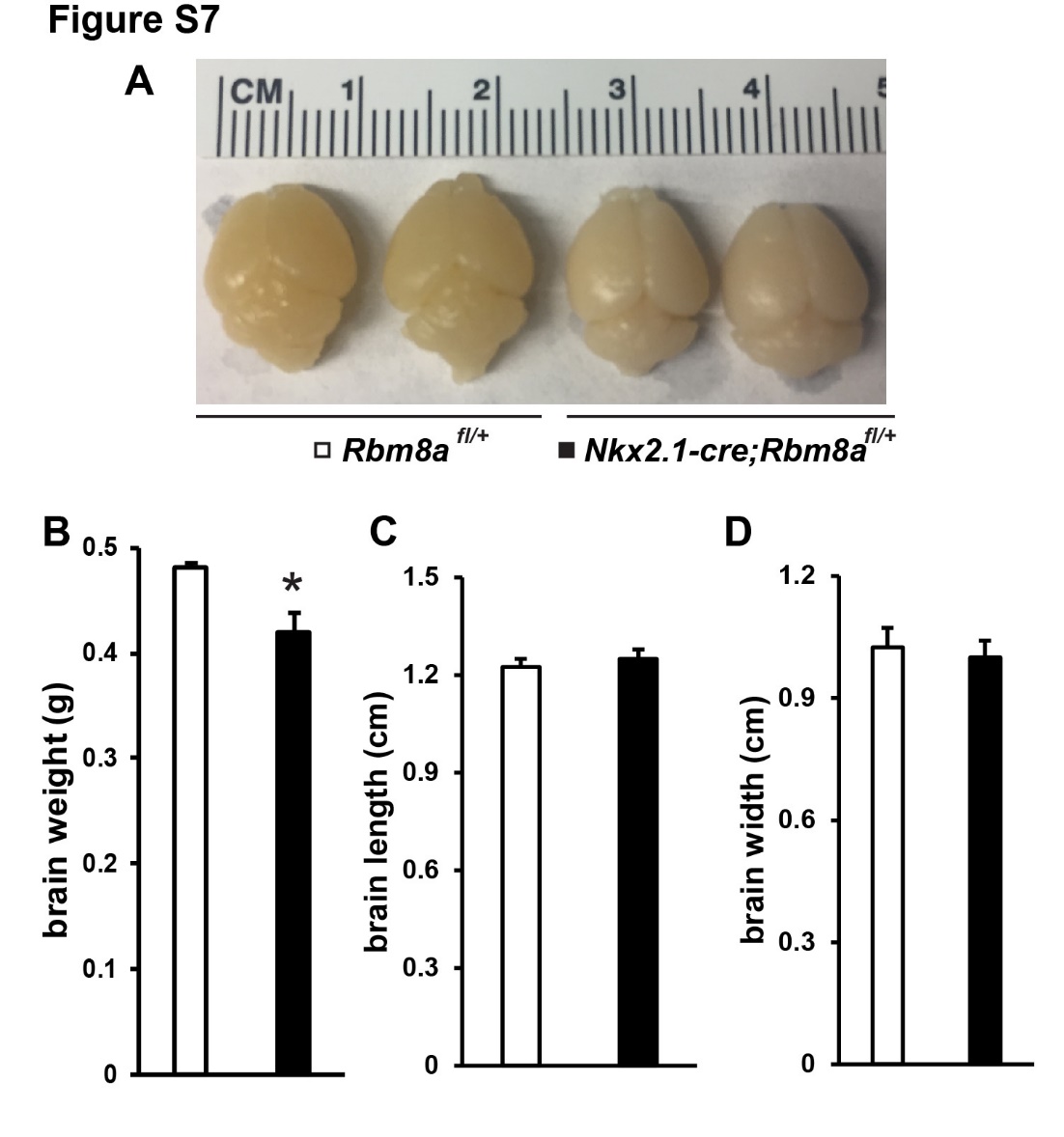


S7. (A) Brains are dissected from *Rbm8a^fl/+^* and *Nkx2.1-cre; Rbm8a^fl/+^* mice at P17 to show the actual brain sizes. (B) Brain weight is slightly decreased in *Nkx2.1-cre; Rbm8a^fl/+^* mice. (C-D) Brain length and width are not significantly changed. *, *p*<0.05, n=4, students t-test.


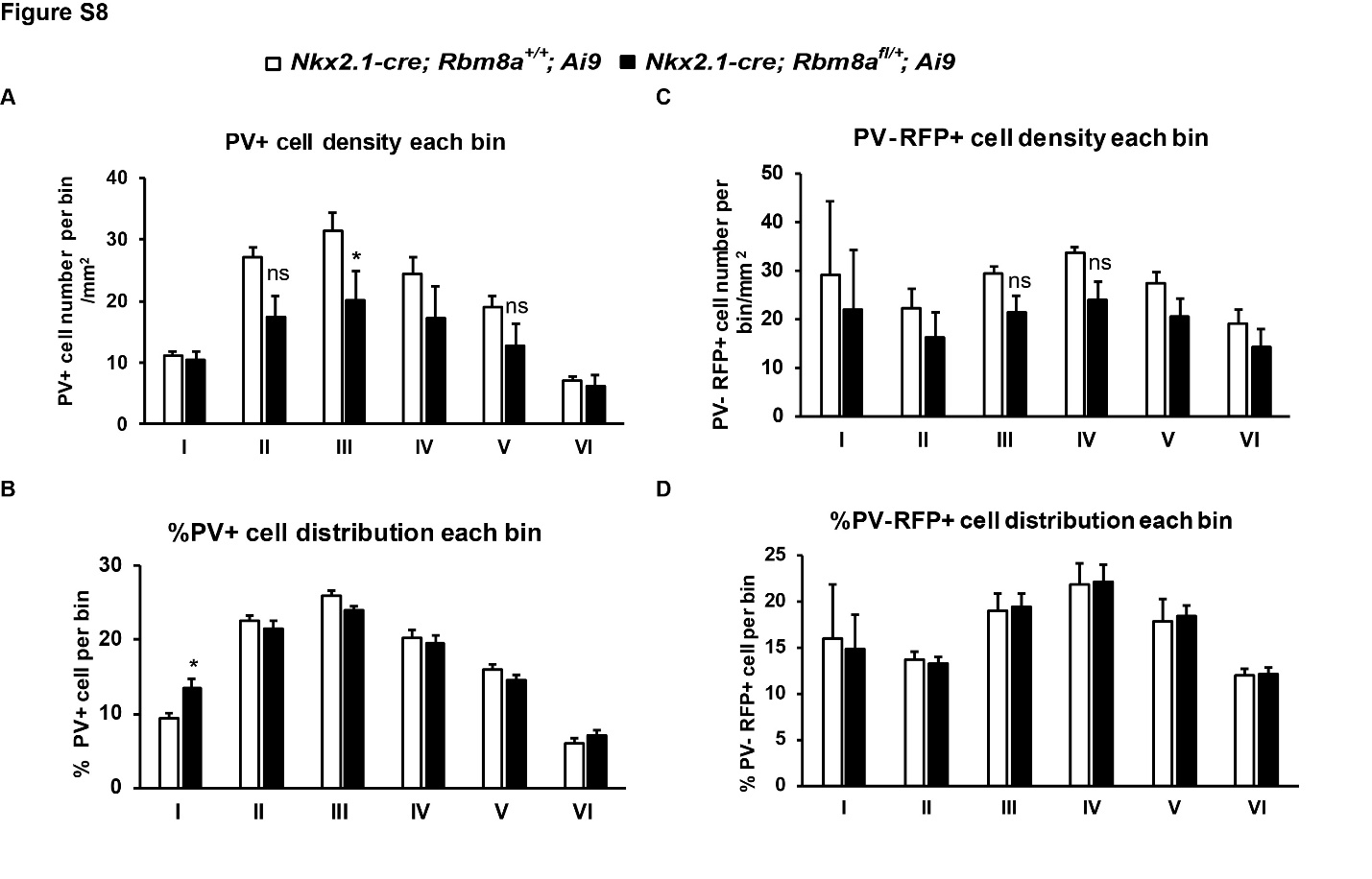


S8. (A) Quantification of PV+ cell density in each bin of the DLPFC of *Nkx2.1–cre; Rbm8a^+/+^; Ai9* and *Nkx2.1–cre; Rbm8a^fl/+^; Ai9* mice. The bar graph shows the PV+ cell number in each bin divided by area. (B) Quantification of percentages of PV+ cells in each bin of the DLPFC. The bar graph shows the percentage of RFP+ cells in each bin to total PV+ cells in all bins. (C) Quantification of PV- RFP+ cell density in each bin of the DLPFC of *Nkx2.1–cre; Rbm8a^+/+^; Ai9* and *Nkx2.1–cre; Rbm8a^fl/+^; Ai9* mice. (D) Quantification of percentages of PV-RFP+ cells in each bin of the DLPFC. *, *p*<0.05; t-test, n=3. Bar graphs represent means +/- SEMs. ns, not significant.


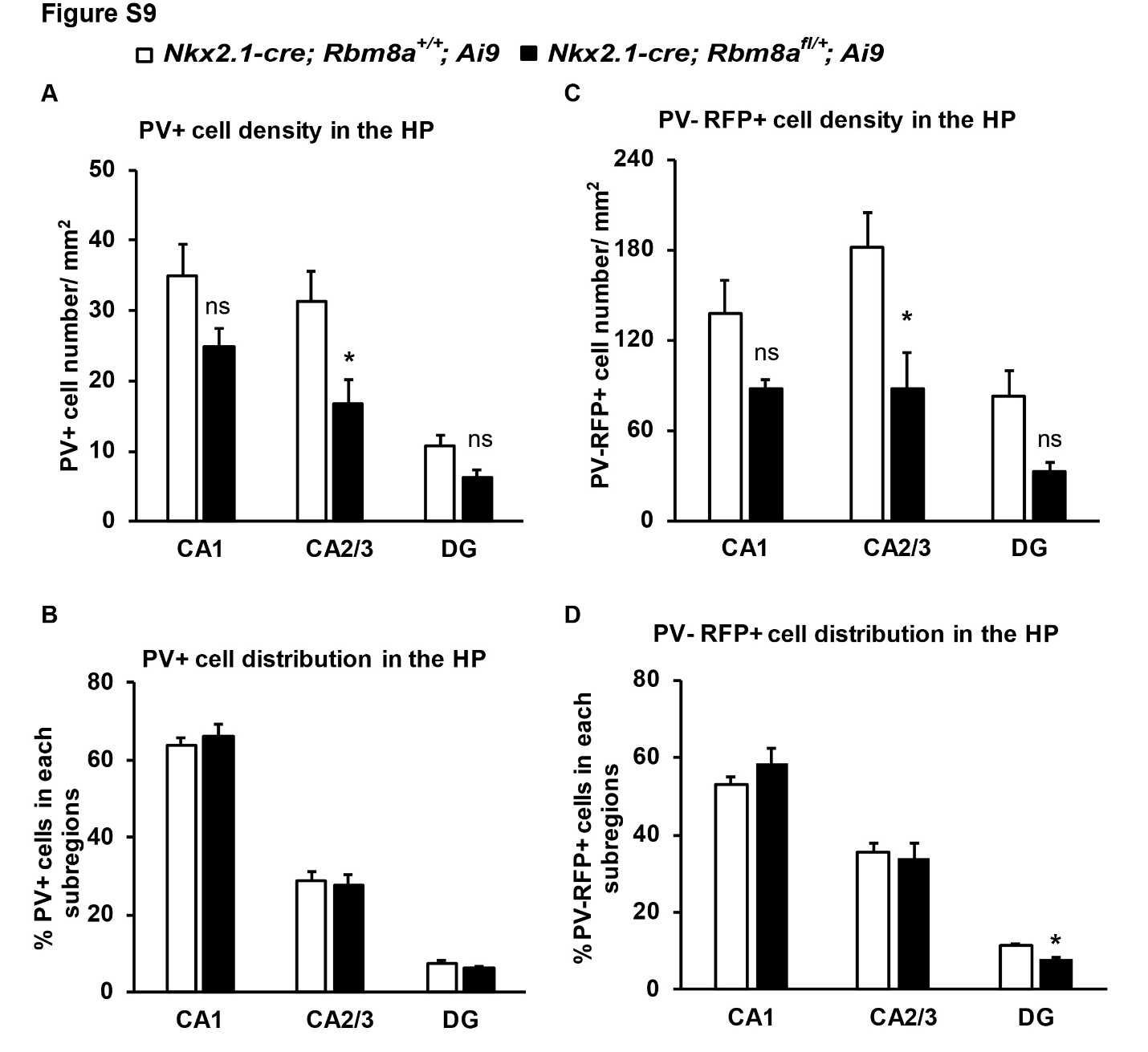
S9. (A) Quantification of PV+ cell density in subregions of the HP. The bar graph shows the PV+ cell number in each subregion divided by area. (B) Quantification of percentages of PV+ cells in each subregion of the HP. The bar graph shows the percentage of PV+ cells in each subregion to total PV+ cells in the whole HP. (C) Quantification of PV-RFP+ cell density in subregions of the HP. (D) Quantification of percentages of PV-RFP+ cells in each subregion of the HP. *, *p*<0.05; t test, n=3. Bar graphs represent means +/- SEMs. ns, not significant.

Table S1: CNS-related DEGs observed in the cortex of E12 *Rbm8a* cKO mice.

19 out of 87 DEGs in the E12 cortex were identified to be relevant to CNS development or function and have fold changes of two or greater. Their names, fold changes, and pathways are listed.

| **Gene ID** | **Gene name** | **Fold change** | **Pathway** |
| --- | --- | --- | --- |
| Abcd2 | ATP-binding cassette D2 | 0.462 | ABC transporter; imports very long fatty acyl-CoA chains into peroxisomes. Likely involved in maintaining the myelin sheath. ^1^ |
| Top2a | Topoisomerase II alpha | 0.518 | A DNA topoisomerase highly expressed in embryonic CNS, especially in rapidly-proliferating cells. ^2^ |
| Fam212b (Inka2) | Inka box actin regulator 2 | 1.278 | Expressed at fluctuating levels throughout development. Associated with proliferating NPCs, some immature neuronal subtypes, and terminally-differentiated neurons in limited areas of the forebrain.^3^ |
| Pcdh11x | Protocadherin 11 X-linked | 1.912 | The X chromosome copy of Pcdh11. The protocadherin pair Pcdh11x/y appears to influence asymmetrical development of the human cerebrum. ^4^ |
| Caly | Calcyon neuron-specific vesicular protein | 2.059 | Binds clathrin, localized in synapses and other membranes, regulated endo/exocytosis. Likely affects neurotransmitter release.^5^ |
| Sst | Somatostatin | 2.139 | A metabolic hormone. In interneurons, Sst regulates synaptic plasticity and excitability.^6^ |
| Hpcal4 | Hippocalcin-like 4 | 2.143 | Binds Ca^2+^; extremely biased expression in the adult cortex and frontal lobe. ^7^ |
| Etv4 | Ets variant 4 | 2.235 | Necessary for dendritic development in some neuronal populations. ^8^ |
| Mal2 | Mal, T cell differentiation protein 2 | 2.300 | A proteolipid in myelin that regulates protein distribution in the membrane.^9^ |
| Fam19a1 | TAFA chemokine like family 1 | 2.361 | Function uncertain. High, biased expression throughout the CNS.^10^ |
| Sncb | Synuclein beta | 2.374 | Synucleins participate in neurotransmitter turnover processes at synapses. Deficiencies can cause decreased dopamine levels and loss of motor control in older mice.^11^ |
| Diras2 | DIRAS family, GTP-binding Ras-like 2 | 2.415 | A Ras GTPase, highly expressed in adult cerebellum.^12^ |
| Vsnl1 | Visinin-like 1 | 2.433 | A neuron-specific calcium sensor protein that associates with the membrane ^13^ |
| Vgf | VGF nerve growth factor inducible | 2.653 | Overexpression of this neuropeptide causes hyperactivity, memory deficits, and other mental symptoms in mice. ^14^ |
| Caln1 | Calneuron 1 | 2.674 | Similar to calmodulin family; localizes to the Golgi membrane and acts as a calcium sensor in the cytoplasm. ^15^ |
| Thy1 | Thymus cell antigen 1, theta | 2.814 | In the CNS, Thy1 marks a subpopulation of neurons in the basolateral amygdala that moderates fear response. ^16^ |
| Zfp365 | Zinc finger protein 365 | 2.863 | Proposed to be a surveyor of telomere dysfunction that prevents chromosomal recombination when telomeres are critically short. ^17^ |
| Nrgn | Neurogranin | 2.986 | Binds calmodulin and CaMKII, active at synapses, participates in synaptic plasticity. ^18^ |
| Kcnc4 | K^+^ voltage gated channel, Shaw-related subfamily 4 | 3.340 | Voltage-gated K^+^ channel; rectifier channel that repolarizes the neuronal axon after an action potential. Subunit composition determines its effects on synaptic excitability. ^19^ |
| Gda | Guanine deaminase | 3.776 | Promotes microtubule assembly; promotes dendritic branching in neurons. ^20^ |

**References:**

1. Fourcade, S.*, et al.* Thyroid Hormone Induction of the Adrenoleukodystrophy-Related Gene (*ABCD2*). *Molecular Pharmacology* **63**, 1296-1303 (2003).

2. Harkin, L. F.*, et al.* Distinct expression patterns for type II topoisomerases IIA and IIB in the early foetal human telencephalon. *Journal of Anatomy* **228**, 452-463 (2016).

3. Iwasaki, Y., Yumoto, T. and Sakakibara, S.-i. Expression profiles of inka2 in the murine nervous system. *Gene Expression Patterns* **19**, 83-97 (2015).

4. Priddle, T. H. and Crow, T. J. The protocadherin 11X/Y (PCDH11X/Y) gene pair as determinant of cerebral asymmetry in modern Homo sapiens. *Annals of the New York Academy of Sciences* **1288**, 36-47 (2013).

5. Muthusamy, N.*, et al.* Phylogenetic Analysis of the NEEP21/Calcyon/P19 Family of Endocytic Proteins: Evidence for Functional Evolution in the Vertebrate CNS. *Journal of Molecular Evolution* **69**, 319 (2009).

6. Liguz-Lecznar, M., Urban-Ciecko, J. and Kossut, M. Somatostatin and Somatostatin-Containing Neurons in Shaping Neuronal Activity and Plasticity. *Frontiers in Neural Circuits* **10**, (2016).

7. Alvaro, C. G.*, et al.* Hippocalcin-like 4, a neural calcium sensor, has a limited contribution to pain and itch processing. *PLOS ONE* **15**, e0226289 (2020).

8. Fontanet, P. A., Ríos, A. S., Alsina, F. C., Paratcha, G. and Ledda, F. Pea3 Transcription Factors, Etv4 and Etv5, Are Required for Proper Hippocampal Dendrite Development and Plasticity. *Cerebral Cortex* **28**, 236-249 (2016).

9. Bijlard, M.*, et al.* MAL Is a Regulator of the Recruitment of Myelin Protein PLP to Membrane Microdomains. *PLOS ONE* **11**, e0155317 (2016).

10. Lei, X.*, et al.* FAM19A1, a brain-enriched and metabolically responsive neurokine, regulates food intake patterns and mouse behaviors. *The FASEB Journal* **33**, 14734-14747 (2019).

11. Connor-Robson, N., Peters, O. M., Millership, S., Ninkina, N. and Buchman, V. L. Combinational losses of synucleins reveal their differential requirements for compensating age-dependent alterations in motor behavior and dopamine metabolism. *Neurobiology of Aging* **46**, 107-112 (2016).

12. Grünewald, L.*, et al.* Expression of the ADHD candidate gene Diras2 in the brain. *Journal of Neural Transmission* **125**, 913-923 (2018).

13. Spilker, C.*, et al.* The neuronal EF-hand calcium-binding protein visinin-like protein-3 is expressed in cerebellar Purkinje cells and shows a calcium-dependent membrane association. *Neuroscience* **96**, 121-129 (2000).

14. Mizoguchi, T.*, et al.* Behavioral abnormalities with disruption of brain structure in mice overexpressing VGF. *Scientific Reports* **7**, 4691 (2017).

15. McCue, H. V., Burgoyne, R. D. and Haynes, L. P. Membrane targeting of the EF-hand containing calcium-sensing proteins CaBP7 and CaBP8. *Biochemical and Biophysical Research Communications* **380**, 825-831 (2009).

16. McCullough, K. M.*, et al.* Molecular characterization of Thy1 expressing fear-inhibiting neurons within the basolateral amygdala. *Nature Communications* **7**, 13149 (2016).

17. Zhang, Y.*, et al.* ZNF365 Promotes Stability of Fragile Sites and Telomeres. *Cancer Discovery* **3**, 798-811 (2013).

18. Díez-Guerra, F. J. Neurogranin, a link between calcium/calmodulin and protein kinase C signaling in synaptic plasticity. *IUBMB Life* **62**, 597-606 (2010).

19. Rowan, M. J. M. and Christie, J. M. Rapid State-Dependent Alteration in Kv3 Channel Availability Drives Flexible Synaptic Signaling Dependent on Somatic Subthreshold Depolarization. *Cell Reports* **18**, 2018-2029 (2017).

20. Akum, B. F.*, et al.* Cypin regulates dendrite patterning in hippocampal neurons by promoting microtubule assembly. *Nature Neuroscience* **7**, 145-152 (2004).
